# Animal Mitochondria, Positive Selection and Cyto-Nuclear Coevolution: Insights from Pulmonates

**DOI:** 10.1371/journal.pone.0061970

**Published:** 2013-04-19

**Authors:** Aristeidis Parmakelis, Panayiota Kotsakiozi, David Rand

**Affiliations:** 1 Department of Ecology and Taxonomy, Faculty of Biology, National and Kapodistrian University of Athens, Athens, Greece; 2 Department of Animal and Human Physiology, Faculty of Biology, National and Kapodistrian University of Athens, Athens, Greece; 3 Department of Ecology and Evolutionary Biology, Brown University, Providence, Rhode Island, United States of America; International Atomic Energy Agency, Austria

## Abstract

Pulmonate snails have remarkably high levels of mtDNA polymorphism within species and divergence between species, making them an interesting group for the study of mutation and selection on mitochondrial genomes. The availability of sequence data from most major lineages – collected largely for studies of phylogeography - provides an opportunity to perform several tests of selection that may provide general insights into the evolutionary forces that have produced this unusual pattern. Several protein coding mtDNA datasets of pulmonates were analyzed towards this direction. Two different methods for the detection of positive selection were used, one based on phylogeny, and the other on the McDonald-Kreitman test. The cyto-nuclear coevolution hypothesis, often implicated to account for the high levels of mtDNA divergence of some organisms, was also addressed by assessing the divergence pattern exhibited by a nuclear gene. The McDonald-Kreitman test indicated multiple signs of positive selection in the mtDNA genes, but was significantly biased when sequence divergence was high. The phylogenetic method identified five mtDNA datasets as affected by positive selection. In the nuclear gene, the McDonald-Kreitman test provided no significant results, whereas the phylogenetic method identified positive selection as likely present. Overall, our findings indicate that: 1) slim support for the cyto-nuclear coevolution hypothesis is present, 2) the elevated rates of mtDNA polymorphims and divergence in pulmonates do not appear to be due to pervasive positive selection, 3) more stringent tests show that spurious positive selection is uncovered when distant taxa are compared and 4) there are significant examples of positive selection acting in some cases, so it appears that mtDNA evolution in pulmonates can escape from strict deleterious evolution suggested by the Muller’s ratchet effect.

## Introduction

MtDNA loci are the most commonly used markers in molecular studies of animal populations [1]. Studies involving the genetic structure of populations, phylogenetic relationships of animal taxa and the spatial and temporal evolution of populations, often involve mtDNA haplotyping. The reasons for the adoption of mtDNA as the favorable marker for molecular studies are well-known (reviewed in [1]). Among other biological attributes, mtDNA is considered to be evolving in a nearly neutral fashion. Being involved in basic metabolic functions (respiration), mitochondrial-encoded genes have been considered as less likely than other genes to be adaptively evolving. However, the fundamental property of the neutral evolution of the mtDNA genome has been questioned [1].

Numerous cases exist [2], [3], [4], [5], [6], [7], [8], [9], [10] in favor of the view that the variation of the mitochondrial genome is not selectively neutral. Additionally, positive selection has been implicated in shaping the mitochondrial diversity recorded in several animal species [5], [6], [7], [10], [11], [12], [13], [14], [15] including humans [11], [16], whereas arguments in favor of purifying selection are also expressed [17]. Fitness consequences [18] are pertinent to the mitochondrial genetic variation that exists within and between populations, and mechanisms through which sequence variation in mtDNA is achieved [19] and maintained [20], [21] have been proposed. Therefore, as it has been concluded [22], “the mtDNA genome is a profound player in the evolutionary dynamics of populations, and the existence of non-neutral genetic variation in mtDNA must be a common phenomenon”.

Aiming at the investigation of the mode of evolution of the mtDNA genome in animal species, we focused on land-snails, a group of organisms that exhibit extreme levels of mtDNA divergence compared to other animal groups [23], [24], [25], [26]. Several researchers have attempted to assess the relative importance of each of the four not mutually exclusive hypotheses that were proposed [23] in order to explain the extreme intra-specific mtDNA divergence observed in pulmonates [23]. These hypotheses are: (i) mtDNA evolution in pulmonates is exceptionally fast, (ii) haplotypes differentiate in isolated ‘refuges’ and subsequently come together, (iii) natural selection is acting to preserve variation, and (iv) the population structure of pulmonates favors the persistence of ancient haplotypes. The accelerated mtDNA evolution hypothesis was supported by the findings in some taxa [24], [26], [27], [28], but not in others [29], [30], [31], whereas a hypothesis was formulated, on developmental grounds, to account for the extraordinary mtDNA diversity in mollusks [32]. At the same time, several studies concluded that a history of allopatric divergence followed by secondary contact (hypothesis ii) could explain sympatry of diverse haplotypes [24], 30,31,33,34,35,36. The effect of natural selection in shaping the population genetic structure of pulmonates (hypothesis iii) has not been directly investigated, but there were cases where natural selection was considered to have played a major role [33], [37], [38]. Furthermore, the potential importance of localized adaptations was demonstrated in *Cepaea* mtDNA [6]. Finally, in some studies it was shown that demographic factors (hypothesis iv), such as highly structured populations and limited capacity for active dispersal, are important both in conservation of mtDNA polymorphisms at a small-scale [39], [40] and in the long-term and large-scale structuring of populations [28], [30], [37], [41]. Moreover, it has been proposed [23], [40] that even in the face of adequate gene flow within demographically structured pulmonate populations, natural selection could favor specific associations between mtDNA and nuclear genes (associations developed whilst populations are separated) and that would radically slow the homogenization process of the mtDNA lineages, thus allowing several divergent mtDNA haplotypes to be maintained within a single pulmonate population.

The hypothesis of natural selection acting on the combined cyto-nuclear genotype, is continuously gaining support [42], [43] as one of the mechanisms through which mtDNA diversity is maintained both within and between animal populations. In this hypothesis, the nuclear genes that interact with the mtDNA genes involved in oxidative phosphorylation (OXPHOS), are the ones more likely to be evolving under the effect of positive selection [42]. These genes are posited to be under a selective regime to maintain functionality of mitochondrial enzyme complexes, driven by the continued loss of function in mtDNA-encoded proteins that experience higher deleterious substitution rates due to reduced effective population size and low recombination rates. Given a high mutation rate in mtDNA, nuclear genes that encode interacting proteins may be more likely to provide positive, compensatory mutations that restore function to OXPHOS complexes that experience deleterious mtDNA mutations [42].

The high levels of mtDNA polymorphism and divergence in pulmonates – and the availability of sequence data from most major lineages – provide a powerful dataset for testing several hypotheses about selection in mtDNA. In an effort to evaluate the role of selection in shaping mtDNA diversity of pulmonates, all the protein coding mtDNA sequences of several pulmonates available in GenBank were analyzed along with sequence data newly generated. The effect of natural selection was investigated through i) extensive phylogenetic analyses, and ii) the implementation of the McDonald-Kreitman test [44]. Additionally, in order to investigate the cyto-nuclear coevolution hypothesis that is proposed to account for the high levels of intra- and inter-population mtDNA divergence, we assessed the divergence pattern exhibited by a nuclear gene (part of the OXPHOS), in two land-snail genera. The nuclear gene investigated is cytochrome c (*Cyc*), a gene that has been identified previously as exhibiting signs of molecular adaptation [18], [45], [46], [47].

Phylogenetic methods are widely used to estimate the evolutionary rates of genes and genomes and to detect footprints of natural selection [48]. To examine whether selection is acting on any of the mtDNA genes investigated, a phylogenetic method to infer the pattern of selection was applied. This method detects elevated dN/dS ratios (ω) under specific models of codon substitution, using maximum likelihood approaches. When the sequences of the reconstructed phylogenetic tree evolve under neutrality, the ratio (also known as ω) of non-synonymous (dN) to synonymous (dS) substitutions is expected to be 1. In the case of positive selection, amino acid changes are favored and ω >1, whereas under purifying selection (i.e. fewer amino acid substitutions than expected by chance) amino acid changes are prevented and ω <1. A different test widely used to infer positive selection is the McDonald-Kreitman test [44] that compares the dN/dS ratio between species to that within species and is based on the neutral assumption that this ratio is the same for polymorphism and divergence data. Under positive selection the dN/dS ratio should be larger between species due to excess fixation of amino acid substitutions. Under purifying selection the dN/dS ratio should be larger within species due to an excess of mildly deleterious amino acid polymorphisms that do not reach high frequencies in the population.

## Materials and Methods

### mtDNA Sequence Data Retrieved from GenBank and Generated for this Study

Data deposited in GenBank up to October 2009 were used. An exception to this is a part of the dataset of *Codringtonia* (see Table 1) that we recently deposited in GenBank. Only coding mtDNA gene sequences of pulmonates were used. The criterion for a specific genus or species to be included in the analysis was that at least seven sequences for at least one coding mtDNA gene should be available. These sequences could originate from different species of the genus (genus analysis) or from different populations of a single species (single species analysis). Sequence datasets were compiled according to genus and mtDNA locus, with the help of CodonCode Aligner v.2.0.6 (CodonCode Corporation, Dedham, MA, USA). The genera analyzed per mtDNA gene are presented in Table 1. In some of the published datasets, the authors concluded that based on the molecular data, species revisions were necessary. In these cases extreme caution was taken to allocate each sequence to the species or genus claimed by the authors (but see further below).

**Table 1:** The pulmonate genera and genes analyzed under the maximum likelihood approach inference of adaptive evolution using PAML.

Gene	Genus	Number of species	Length of alignment/Number of congeneric sequences in alignment	Reference/GenBank Accession Numbers	McDonald-Kreitman test (Y/N)
***COX1***					
	*Achatinella*	10	675/77	[87], [88]	Y
	*Agathylla*	11	729/29	see supporting information file	Y
	Albinaria1@	9	651/11	[89], [90], [91]	N
	Albinaria2@	9	800/40	see supporting information file	Y
	*Amborhytida*	6	948/36	[92]	Y
	*Arianta*	4	594/64	[91], [93]	Y
	*Arion* *	25/1	549/38	[94], [95], [96]	Y
	*Ashmunella*	5	597/42	Unpublished: AY823787-AY823831	Y
	*Bulimulus*	23	450/87	[97]	Y
	*Candidula*	7	522/11	[91], [98]	Y
	*Carinigera*	10	627/31	[90], [99]	Y
	*Codringtonia* *	8/1	702/83	[84]; see supporting information file	Y
	*Elona* **	1	681/36	[100]	Y
	*Euchemotrema* **	1	648/12	[101]	Y
	*Euhadra*	15	552/77	[34], [102]	Y
	*Everettia*	14	558/46	[103], [104]	Y
	*Iberus*	9	627/85	[105], [106], [107]	Y
	*Isabellaria*	14	651/24	[89], [90]	N
	*Kovacsia* **	1	654/21	[108]	Y
	*Leptaxis*	5	375/9	[109]	N
	*Lozekia* **	1	654/17	[108]	Y
	*Marmorana*	7	597/33	[110]	Y
	*Natalina*	12	978/62	[111]	Y
	*Oreohelix*	7	687/35	[112]	Y
	*Partula*	11	663/146	[113], [114], [115]	Y
	*Partulina*	12	675/29	[88]	Y
	*Paryphanta* **	1	948/15	[92]	Y
	*Placostylus*	6	672/20	[115], [116]	Y
	*Prophysaon*	8	618/43	[117]	N
	*Pupilla*	2	654/10	[118]	Y
	*Satsuma* *	25/1	666/183	[89], [90], [119]	Y
	*Sericata*	12	627/24	[89], [90]	Y
	*Solatopupa*	7	342/14	[120]	Y
	*Succinea*	14	612/61	[115], [121]	Y
	*Trochulus*	10	558/97	[122]	Y
	*Vertigo*	21	654/65	[123]	Y
	*Wainuia*	6	414/21	[92], [124]	Y
	*Xerocrassa*	11	633/122	[125]	Y
***COX2***					
	*Agathylla*	11	549/29	see supporting information file	Y
	*Codringtonia* *	8/1	558/82	[84]	Y
	*Gnarosophia* **	1	480/41	[126]	Y
	*Sphaerospira*	7	465/58	[126]	Y
***Cytb***					
	*Euhadra*	19	726/63	[127], [128]	Y
	*Partula*	19	633/29	[53]	N
	*Pupilla*	2	396/8	[118]	Y
***ATPase 8***					
	*Albinaria1* *	3/1	165/32	[129]	Y
	*Cepaea* **	1	153/32	[6]	Y
***ND1***					
	*Arion*	20	435/40	[130]	Y
	*Euhadra*	19	261/63	[127]	Y
***ND4L***					
	*Euhadra*	19	255/63	[127]	Y
***Cyc***					
	*Albinaria2*	9	249/19	see supporting information file	Y
	*Codringtonia*	6	249/19	see supporting information file	Y

* PAML analyses performed both on multiple species and on a single species (multiple populations per species).

** PAML analyses performed separately on single species of the genus (multiple populations per species).

@ The two datasets of Albinaria in COX1 include different species.

Length of sequences is given in base pairs (bp). The datasets that were subjected to the McDonald-Kreitman test are indicated with Y. Accession numbers are provided for sequence data generated for this study, and for sequence data available in GenBank but not published.

Besides the mtDNA data retrieved from GenBank, we also analyzed unpublished mtDNA sequences (two genes) generated for the present study, originating from one additional pulmonate genus. This genus is *Agathylla* (Westerlund, 1898) (Table 1 and Table S1 in File S1). Furthermore, we analyzed several unpublished *COX1* sequences of *Codringtonia* Kobelt, 1898 (Table 1 and Table S1 in File S1) a genus already represented in GenBank records. Moreover, we generated and analyzed (Table 1 and Table S1 in File S1) mtDNA sequences for *Albinaria* Vest, 1867 that is represented in GenBank but with a different set of species. Details regarding DNA extraction methods, PCR amplification and sequencing of the mtDNA fragments in *Agathylla*, *Albinaria* and *Codringtonia* are provided in the supporting information file (File S1). Accession numbers of generated sequences are provided in Table 1 and Table S1 in File S1.

In the *COX1* sequences, in total 37 genera of pulmonates were represented, whereas in the remaining mtDNA genes 1–4 genera were represented. An overview of the genera analyzed is provided and the reference from which the data originate is cited in Table 1.

Basic analyses of polymorphism and divergence were performed using the software DNAsp v.5.10 [49] and MEGA v.4.0 [50]. The estimated parameters were the number of non-synonymous substitutions per non-synonymous site (dN or Ka) and the number of synonymous substitutions per synonymous site (dS or Ks). Two additional parameters estimated were the “net between group average” level of sequence divergence between species and the mean intra-species divergence. These parameters were estimated separately for each gene and genus, using MEGA v.4.0 and the Kimura two-parameter distance [51]. For both the mtDNA and the nuclear gene an additional measure of sequence divergence is presented in Table 2. This divergence metric is the tree length (nucleotide substitutions per codon along the tree), and was estimated by Codeml (model M0) of the PAML package [52].

**Table 2:** Likelihood Ratio (LRTs) and McDonald-Kreitman tests' results on the sequence datasets of the mtDNA genes *COX1, COX2, Cytb, ND1, ND4L, ATP8* and the nuclear gene *Cyc* of pulmonates.

Gene	Pulmonate genus/species	TL	LRT p-value significance in model comparison			Number of amino acids with dN/dS >1	Mc-Donald Kreitman test p-value^a^ (npc)	FDR correction for multiple hypothesis testing	Total number of estimable N.I. values
			M0 vs. M3	M1a vs. M2a	M7 vs. M8				
***COX1***	*Achatinella*	2.23	**	n.s.	n.s.	−	n.s.		36
	*Agathylla*	18.4	**	n.s.	n.s.	−	* (1) ** (2)	(3)	9
	*Albinaria1*	7.45	n.s.	n.s.	n.s.	−	not performed		−
	*Albinaria2*	10.12	n.s.	n.s.	n.s.	−	n.s.		5
	*Amborhytida*	17.92	n.s.	n.s.	n.s.	−	n.s.		1
	*Arianta*	4.84	n.s.	n.s.	n.s.	−	n.s.		51
	*Arion*	34.29	n.s.	n.s.	n.s.	−	* (4) ** (9)	(9)	89
	*Ashmunella*	3.11	n.s.	n.s.	n.s.	−	n.s.		6
	*Bulimulus*	7.33	n.s.	n.s.	n.s.	−	n.s.		119
	*Candindula*	8.85	n.s.	n.s.	n.s.	−	n.s.		3
	*Carinigera*	7.48	n.s.	n.s.	n.s.	−	n.s.		8
	*Codringtonia*	6.5	**	n.s.	n.s.	−	n.s.		12
	*Elona quimperiana*	0.014	n.s.	n.s.	n.s.	−	n.s.		0
	*Euchemotrema hubrichti*	0.05	n.s.	n.s.	n.s.	−	n.s.		0
	*Euhadra*	19.6	n.s.	n.s.	n.s.	−	n.s.		24
	*Everettia*	12.97	n.s.	n.s.	n.s.	−	n.s.		10
	*Iberus*	16.03	n.s.	n.s.	n.s.	−	n.s.		55
	*Isabellaria*	18.58	n.s.	n.s.	n.s.	−	not performed		−
	*Kovacsia kovacsii*	0.59	n.s.	n.s.	n.s.	−	n.s.		0
	*Leptaxis*	4.2	n.s.	n.s.	n.s.	−	not performed		−
	*Lozekia deubeli*	1.35	**	n.s.	n.s.	−	n.s.		15
	*Marmorana*	5.5	n.s.	n.s.	n.s.	−	n.s.		0
	*Natalina*	26.03	n.s.	n.s.	n.s.	−	* (7) ** (28)	(31)	55
	*Oreohelix*	2.34	*	n.s.	n.s.	−	n.s.		12
	*Partula*	10.72	n.s.	n.s.	**	−	* (1)	(0)	28
	*Partulina*	1.38	**	n.s.	n.s.	−	n.s.		7
	*Paryphanta busbyi*	0.18	n.s.	n.s.	n.s.	−	n.s.		1
	*Placostylus*	14.78	**	n.s.	**	−	n.s.		3
	*Prophysaon*	11.25	n.s.	n.s.	n.s.	−	not performed		−
	*Pupilla*	5.55	**	n.s.	n.s.	−	n.s.		1
	*Satsuma*	30.96	n.s.	n.s.	n.s.	−	* (4)	(0)	88
	*Sericata*	19.18	n.s.	n.s.	**	−	n.s.		3
	*Solatopupa*	3.71	n.s.	n.s.	n.s.	−	n.s.		2
	*Succinea*	8.69	n.s.	n.s.	n.s.	−	n.s.		3
	*Trochulus*	9.56	n.s.	n.s.	n.s.	−	n.s.		63
	*Vertigo*	14.95	n.s.	n.s.	n.s.	−	n.s.		23
	*Wainuia*	0.81	*	n.s.	n.s.	−	n.s.		5
	*Xerocrassa*	13.18	**	n.s.	**	−	* (1)	(0)	39
***COX2***	*Agathylla*	15.44	**	n.s.	n.s.	−	* (4) ** (3)	(5)	15
	*Codringtonia*	5.96	**	n.s.	n.s.	−	** (1)	(0)	20
	*Gnarosophia bellendenkerensis*	2.08	**	n.s.	n.s.	−	n.s.		25
	*Sphaerospira*	12.69	n.s.	n.s.	n.s.	−	n.s.		15
***Cytb***	*Euhadra*	15.22	n.s.	n.s.	n.s.	−	* (31) ** (28)	(25)	189
	*Partula*	8.74	**	n.s.	*	−	not performed		−
	*Pupilla*	0.66	n.s.	n.s.	n.s.	−	n.s.		1
***ND1***	*Arion*	33.83	**	n.s.	n.s.	−	* (12) ** (40)	(50)	77
	*Euhadra*	19.64	**	n.s.	n.s.	−	* (10) ** (2)	(0)	206
***ND4L***	*Euhadra*	18.99	**	n.s.	n.s.	−	* (25) ** (8)	(0)	157
***ATP8***	*Albinaria*	2.53	**	n.s.	n.s.	7^c^	n.s.		4
	*Cepaea nemoralis*	1.26	**	n.s.	n.s.	8^c^	n.s.		12
***Cyc***	*Albinaria2*	0.14	**	*	*	1^b^, 2^c^	n.s.		2
	*Codringtonia*	0.69	**	n.s.	n.s.	1^b^	n.s.		5

* p<<0.05,

** p<<0.01, n.s.: non significant,

a Fisher' s test p-value, npc: number of pairwise comparisons that the neutrality index (N.I.) was <1, FDR: False Discovery Rate for multiple-hypothesis-testing correction,

b indicated (probability >0.99) both by the Bayes empirical Bayes and the Naïve empirical Bayes methods in all model comparisons,

c indicated only by the Naïve empirical Bayes method in all model comparisons.

The tree length (TL) is given as a measure of sequence divergence (nucleotide substitutions per codon along the tree).

The data used in this work were generated by researchers mostly concerned about the phylogeny of the genus under study. Consequently, it is very possible that even though the majority of the species of a single genus have been included in each study, only a few populations from each species have been used. These conspecific populations could originate from a variety of distant and/or neighboring localities, hence within species level of divergence in some cases might be misleading. Such issues were evaluated on a case-by-case basis and where necessary such cases were excluded from the inference of general patterns.

### Land-snail Genera in which the Cyc Gene was Amplified and Analyzed

The pulmonate genera in which the nuclear gene (*Cyc*) fragment was amplified are *Codringtonia* and *Albinaria* (Table 1). Details on the geographic origin and species status of the specimens used in the study, as well as accession numbers of generated sequences are given in Tables 1 and S1. Details on specimens’ collection, nucleic acids extraction, cDNA synthesis, RT-PCR, and cloning of the *Cyc* amplicons are provided in the supporting information file.

### Investigating Selection Using Maximum Likelihood Analysis (PAML) and the McDonald-Kreitman Test

Extensive description of the alignment of the sequences and the phylogenetic analyses performed, are given in the supporting information file. The generated Bayesian tree of each mtDNA gene and genus (or single species) served as the basis for the implementation of the maximum likelihood methods of the PAML package [52] aiming at detecting adaptive molecular evolution under specific models of codon substitution. In the vast majority of the cases, outgroups were removed (pruned) from the Bayesian tree prior to the maximum likelihood analysis. In order to evaluate the effect of the pruning scheme applied in the PAML analyses, multiple sets were subjected to a Bayesian analysis without involving outgroup sequences and then were re-analyzed with PAML. The results of both PAML analyses regarding the presence or not of positive selection were identical to each other. Furthermore, the dataset of *Cytb* of *Partula* produced a highly unresolved phylogenetic tree. However, in order to obtain a more representative picture for the evolution of *Cytb*, the dataset of *Partula* was subjected to the PAML analysis using as starting tree the already published one [53]. Finally, in the inference of positive selection for the nuclear *Cyc* gene of *Albinaria* and *Codringtonia*, the *COX1* mtDNA tree of the respective specimens was used. This was done to eliminate the effect of recombination [54] in the inference of positive selection in the nuclear sequence data.

In each one of the genes subjected to the PAML analysis, the likelihood values of the respective phylogenetic tree as being the result of lineages evolving under the assumptions of the site models M0, M3, M1a, M2a, M7 and M8, were estimated. These models (except M0) allow ω (dN/dS) values to vary among different codons. In practice, maximum likelihood estimates that assume a single value of ω (model M0) often produce conservative results because positive selection in a small subset of sites can be confounded by the effects of purifying selection at other sites. A way to circumvent this is to assume heterogeneity among sites and thus evaluate models that allow for variation among sites such as the M1a, M2a, M3, M7 and M8. For a more detailed description of the models used and the approach followed see the supporting information file. In PAML the Naïve empirical Bayes (NEB) [55], [56] and the Bayes empirical Bayes (BEB) methods [57] are used to identify sites under positive selection. BEB is implemented under models M2a and M8 only. As suggested in the PAML manual only the results of the BEB method should be considered to be robust in the identification of sites under selection.

In the supporting information file, we are addressing the issue of using recombining sequences in the inference of positive selection through phylogeny. The McDonald-Kreitman test as implemented in DNAsp v.5.10 [49], was applied in all the datasets where feasible (Table 1). In some datasets (e.g. *Cytb* of *Partula*), the McDonald-Kreitman test was not applied, since many of the species were represented by a single specimen. The test was implemented both in pairwise comparisons of different species of a genus and when possible in pairwise comparisons of different populations of a single species. In both cases, the nucleotide sequences of the mtDNA gene under study, were grouped according to their phylogeny (Bayesian tree) and not based on species or population of origin. The neutrality index (N.I.) [58] and the test of Fisher (as implemented in DNAsp v.5.10) were used to evaluate the strength and statistical significance of positive or negative selection indicated by the McDonald-Kreitman test. We considered removing rare single nucleotide polymorphisms (SNPs) from the datasets to see if this would alter the selection patterns inferred by the test. However, we chose not to do so because it is well known that removing low frequency non-synonymous sites will change the McDonald-Kreitman test and N.I. values in the direction of positive selection [59], [60]. Our question here is whether a signature of positive selection is pervasive over-and-above the natural level of mildly deleterious variation that is well known to exist for animal mtDNA [61], [62]. Therefore, the removal of rare singletons that is applied when trying to discover the proportion of sites that are fixed by positive selection [59], is not justified in our study. For the reasons stated above, we only applied the McDonald-Kreitman test and no modifications or improvements of this test were implemented. When several statistical inferences based on the same dataset are performed simultaneously, hypothesis tests that incorrectly reject the null hypothesis are more likely to occur. To cope with this, a stronger level of evidence is required for an individual test to be stated as significant, to compensate for the number of tests being made. We applied the false discovery rate (FDR) analysis [63] to correct for such pseudoreplicated comparisons by computation of a p-value cut-off [64] in those datasets for which the McDonald-Kreitman test was in favor of positive selection acting on the sequences.

It has been argued that in datasets where multiple substitutions at each synonymous site have occurred, the number of synonymous substitutions will probably be underestimated and this can lead to erroneous inference of positive selection [61], [65], [66], [67], [68]. In order to investigate the issue of substitution saturation argument we estimated (in all datasets) the pairwise estimates [69] of dN and dS and plotted them against the respective pairwise genetic distance calculated based on the Jukes-Cantor model [70]. Relationships between the Jukes-Cantor distance and dN and dS were modeled separately using the ordinary least squares method. The cubic, quadratic and the linear function of the Jukes-Cantor distance were tested in order to account for curvilinear relationships. Details on the model fitting procedure are given in the supporting information file.

## Results

### Generated Cyc and mtDNA Sequences

Details on the generated mtDNA sequences from specimens of *Agathylla*, *Albinaria*, and *Codringtonia* are given in Table S1. We successfully amplified a fragment of the *Cyc* gene in 38 land-snail specimens, 19 from each land-snail genus targeted (Table 1). After assigning the individual specimens to the nominal species they belong to, we had *Cyc* sequences for six *Codringtonia* and nine *Albinaria* species, respectively (Table 1). In all specimens a single *Cyc* allele was present. The length of the obtained fragments ranged from 139 to 202 (average 172 bp) and 59 to 250 bp (average 190 bp) for *Albinaria* and *Codringtonia*, respectively.

### Levels of Sequence Divergence between and within Species of Pulmonates

The mean levels of sequence divergence between species (mean inter-specific divergence) and within species (mean intra-specific divergence) for each mtDNA gene and land-snail genus are presented in Figure S1 in File S1. According to the data available, the mean sequence divergence between different species of pulmonates could be in the range of 2% to 34.5% depending on the mtDNA gene used. At the same time the within species sequence divergence is between 0.2 and 13.8%. A more detailed presentation of the divergence levels is provided in the supporting information file.

Thirty-eight sequences of *Cyc* from two land-snail genera were produced for this study. The between-species levels of sequence divergence for *Cyc* range from 0 to 3.1% in *Albinaria* and 0 to 5.8% in *Codringtonia*. The within-species level of sequence divergence is in the range of 0 to 4.3% in *Codringtonia* and 0 to 0.6% in *Albinaria*.

### Detecting Selection Using Maximum Likelihood Analysis and the McDonald-Kreitman Test

All the datasets were subjected to several tests designed to detect recombination (see supporting information file) which was not identified in any of the datasets that provided evidence for positive selection (PAML or McDonald-Kreitman). In Table 2 we are providing the results of the maximum likelihood analysis and the McDonald-Kreitman tests on the available datasets. More detailed results per evaluated gene are presented below.

In total, 41 datasets of *COX1* sequences originating from 37 pulmonate genera (Table 1) were analyzed using maximum likelihood analysis and investigated for the presence of positive selection. Out of the 41 datasets, the LRT in 10 of them (25% of the datasets) indicated that varying selection pressure (M0 versus M3) is acting along the protein (Table 2). However, no amino acids were indicated as evolving under positive selection. The *COX1* datasets were also tested with the McDonald-Kreitman test and in six cases the results were in favor of the presence of positive selection (Table 2). The neutrality index (N.I.) in several pair-wise comparisons within the genera of *Agathylla*, *Arion*, *Natalina*, *Partula*, *Satsuma* and *Xerocrassa* was significantly less than 1 and the p-value of the Fisher’ s test was significant. After correcting for multiple comparisons using the FDR, no support for positive selection was evident in the datasets of *Partula*, *Satsuma* and *Xerocrassa* based on the McDonald-Kreitman test (Table 2).

When the M7 model was evaluated against the M8 model in PAML, four *COX1* datasets of pulmonates out of the 41 (10% of the datasets) were in favor of the M8 model being more appropriate for the specific data (Table 2). Two of these datasets indicated positive selection in the M0 versus M3 model comparison as well. Once more, even though the presence of positive selection is favored by the model comparison, no amino acids were indicated as exhibiting dN/dS values greater than 1.

The M1a versus M2a model test did not produce any significant results supporting positive selection acting on the *COX1* gene in any of the pulmonate datasets. The same is true for all remaining mtDNA genes and therefore we will not be referring to this model comparison for the remaining mtDNA genes.

Five *COX2* gene datasets were analyzed (Table 1). In the maximum likelihood analysis (PAML) three of them were in favor of the M3 model versus the M0 (Table 2). This signifies that there is variation in the selective pressure acting along the protein sequence. On the other hand, no amino acids were identified as being affected by positive selection. The McDonald-Kreitman test performed on *COX2* datasets, produced several significant results supporting the possible role of positive selection in shaping the molecular divergence of the sequences in two land-snail genera (*Agathylla* and *Codringtonia*). After the FDR correction only the dataset of *Agathylla* was still indicating signs of positive selection (Table 2).

Out of the three available *Cytb* datasets of pulmonates, one (*Partula*) exhibited signs of non-uniform selection pressure among the amino acid sites (M0 versus M3). The same result was obtained from the M7 versus M8 test (Table 2). In both model comparisons however, no specific amino acids were recognized as exhibiting dN/dS values above 1. The *Cytb* dataset for *Partula* could not be subjected to the McDonald-Kreitman test since no intra-species data were available. However, the test was implemented in the remaining two *Cytb* datasets and was in favor of positive selection acting on the *Cytb* of *Euhadra*. There were 59 (significantly supported) species pairwise comparisons that returned a N.I. value that was less than 1. After the multiple hypothesis testing correction, a quite high number of pairwise comparisons supporting positive selection (25) was maintained (Table 2).

In total two datasets of pulmonates were available for *ATP8* and both of them were analyzed with PAML and provided evidence of positive selection acting on the ATP8 protein. The evidence was derived only from the M0 versus M3 model comparison (Table 2) and in both datasets several amino acid sites were identified as exhibiting dN/dS values greater than 1. However, these sites were solely identified by the NEB method and not by the BEB method (Table 2). On the other hand, the McDonald-Kreitman test was not in favor of positive selection acting on the protein in any dataset (Table 2).

Two *ND1* datasets were analyzed. Both of them favored the M3 versus the M0 model (Table 2). Once more non-uniform selection pressure acting on the amino acids was identified. The attempt to identify positively selected sites was not successful (Table 2). At the same time, the McDonald-Kreitman test identified 52 cases of pairwise comparisons (total number of estimable N.I.: 77, Table 2) with statistically strong evidence of positive selection in *Arion* and 12 (out of estimable N.I.: 206) in *Euhadra* (Table 2). After the FDR adjustment the number of pairwise comparisons indicating positive selection in *Arion* was 50 and 0 for *Euhadra*.

A single *ND4L* gene dataset (Table 1) was analyzed and the maximum likelihood analyses were not in favor of positive selection acting on this gene. On the other hand, the McDonald-Kreitman test indicated 33 cases (out of estimable N.I.: 157) of pairwise comparisons with strong evidence of the neutrality index being less than 1. Following the FDR correction, no signs of positive selection could be found in this dataset (Table 2).

Signs of positive selection were detected in the *Cyc* dataset of *Albinaria*. All model comparisons were in favor of positive selection, and both the LRT and the BEB analyses supported this finding. The BEB analyses, in all model comparisons, indicated a single amino acid as evolving under the effect of positive selection. In the dataset of *Codringtonia* the LRT results were not in favor of positive selection acting on *Cyc*. However, the BEB analyses strongly supported the presence of a single amino acid as exhibiting adaptation in all model comparisons.

### Mutational Saturation

The results of the three different models fitted to describe the relation of dS and dN with genetic distance (Jukes-Cantor) are presented in the supporting information file. Two detailed tables are provided, one for dS (Table S2 in File S1) and one for dN (Table S3 in File S1) along with the figures (Fig. S2a, S2b, S2c, S3a, S3b, S3c in File S1) of dS and dN versus genetic distance for all 45 land-snail datasets.

In the majority of the datasets for both dS and dN either the cubic or the quadratic model provides a better fit. More specifically, in the dS analyses the cubic model shows a better fit in 30 out of the 45 land-snail datasets, whereas the quadratic is more appropriate in 11 datasets and the linear only in four (Table S2 in File S1, w_i_ values). Moreover, in the 40 datasets in which a polynomial model exhibits a significant better fit, there are 15 cases (Table S2 in File S1) in which a declining trend of dS values (as a function of the genetic distance) is predicted (i.e. negative value of terminal slope of the best polynomial model).

In the dN analyses, based on the w_i_ values it is evident that in 31 out of the 45 datasets, the cubic model has a better fit (Table S3 in File S1). The quadratic is indicated as better fitted in 13 datasets, whereas the linear model only in three. Finally, in these 42 datasets that the dN values are better described as a function of the genetic distance using a polynomial model, there are 12 cases where declining values of dN are predicted (Table S3 in File S1).

The results using the AIC were similar to those using the BIC. There were some cases where a different model was selected by the AIC. More specifically, in seven datasets a more complex model (cubic: 6, quadratic: 1) was selected for dS. Regarding dN there were six datasets for which the more complex cubic model was chosen. However, the substitution trends when different models were preferred remained the same.

Aiming at further investigating the effect of mutational saturation on the inference of selection we compared the tree lengths of datasets showing positive selection in the McDonald-Kreitman tests with those that do not. This comparison was performed using a Mann-Whitney U test (results not shown) and it was clearly indicated that the tree length of the analyzed datasets has a significant impact on the proportion of significant MacDonald-Kreitman tests and this relationship is opposite for datasets showing positive or negative selection. It was evident that the tree length is significantly longer for the datasets showing positive selection than for those that are not showing signs of positive selection, while there is no significant difference between datasets showing negative selection and those that do not deviate from neutrality.

## Discussion

Land-snails are known to exhibit high levels of mtDNA divergence [23], [24], [26]. Figure S1 in File S1 presents the mean levels of inter- and intra-specific sequence divergence based on the Kimura-2p model. According to these data, it is evident that both the intra- and inter-specific levels of divergence are extremely high. Divergence between species is as high as 35%, whereas intra-specific differences rise up to 13.8% (Figure S1 in File S1). The levels of divergence in pulmonates are comparable to those found for homologous sequences within taxa described as genera, families or even orders for other organisms, as has already been stated [23]. Therefore, investigating the mode of evolution of the pulmonate mtDNA is challenging. Given the extremely high level of intra-specific sequence divergence reported here (13.8%, supporting information file), it could be argued that certain mtDNA alleles may have diverged for a long time in allopatry before coming together subsequently and might represent populations that are no longer able to interbreed. Such a situation is likely to produce significant results of the McDonald-Kreitman test in which the N.I. values will be greater than 1 but with polymorphic non-synonymous substitutions occurring at intermediate frequencies in the incriminated population, a situation that is quite different from strong purifying selection in which polymorphic non-synonymous mutations occur at low frequency. However, as we mention in the materials and methods section, the sequences were assigned to phylogenetic clades and not nominal species. This was done to avoid inflating the parameters indicating positive selection when cryptic species are included in such analyses [71]. Furthermore, it has been shown that individuals of a single population diverging as much as 6.3% in their *16S rDNA* sequences can interbreed and exchange spermatophores in the field [25]. Therefore, it cannot be conclusively claimed that individuals from different populations being separated by mtDNA sequences differing as much as 13.8%, cannot interbreed. On the contrary, there are strong indications that they can, but the question remains whether or not they can produce viable offspring.

Other invertebrate groups have also been found to exhibit extreme levels of mtDNA divergence, such as copepods [72] and hymenoptera [13]. In most cases the small effective population size of the mtDNA, is invoked to explain the faster accumulation of mutations in the mitochondrial genome compared to the nucleus. However, researchers are considering several other factors as contributing to this pattern. For instance lineage-specific attributes such as body size [73], generation time [74], effective population size [68], [75], and presence of *Wolbachia* [13], have been considered to affect both the mutation rate exhibited as well as the possibility of a mutation becoming fixed in a population. Pulmonates are ubiquitously hermaphrodites [32] and as such the effective population size of the organelles is in theory twice that of normal (all else being equal), since all individuals in a population can potentially contribute to the next generation [32]. Therefore the smaller effective population size argument is weakened to some degree. As already indicated in the introduction, one of the four competing hypotheses that have been proposed to account for the high levels of intra-specific mtDNA divergence of pulmonates is that of natural selection acting on the mtDNA haplotypes and shaping the genetic structure of pulmonates’ populations. Through the approach followed in the present work, we evaluated the role of natural selection in shaping the diversity of the protein-coding mtDNA sequences of pulmonates. Moreover, we took a step further and investigated the cyto-nuclear coevolution hypothesis that has also been proposed to account for the high levels of intra- and inter-population mtDNA divergence recorded in animals [42], [43].

The inter-specific levels of divergence estimated for *Cyc* in *Albinaria* (0 to 3.1%), are within the range reported for other invertebrate species. However, in *Codringtonia* the values obtained (0 to 5.8%) are higher. In *Tigriopus californicus* [76] the divergence values between different populations (or species according to Willett and Burton [45]) range from 0 to 3.3%, whereas in *Carabus* [77] the inter-specific divergence levels are in the range of 0 to 3.8%. No data are available for intra-specific comparisons.

### Indications of Selection in the mtDNA Genes

Based on the phylogenetic and population genetic analyses performed on the mtDNA sequences datasets, it can be claimed that the signals for positive selection obtained from the two different kinds of analyses are partially congruent. Starting from the McDonald-Kreitman test, it provides several indications of positive selection acting on the mtDNA genes evaluated (Table 2). In a preliminary analysis (results not shown) performed on the same datasets but without implementing the phylogenetic grouping scheme in the analysis (see materials and methods section) the results of the McDonald-Kreitman test were providing additional cases of positive selection. However, we have chosen to define intra- and inter-specific groups based on phylogenetic analyses since according to our view it makes more sense, and furthermore it ensures the escape from the pitfall of cryptic species present within the datasets that may well inflate the parameters indicating positive selection, an issue that was addressed in [71] as well. Additionally, when a correction for multiple hypothesis testing was applied to the results of the McDonald-Kreitman test, the support of positive selection was further reduced (Table 2).

### McDonald-Kreitman Test and Substitution Saturation

In numerous studies [61], [66], [67], [68], [78] it has been argued that the McDonald-Kreitman is prone to exhibit false positives of selection when the mutation saturation level is high. The levels of dN and dS saturation were investigated in the datasets analyzed in the present work. For this analysis we have to acknowledge that only a limited number of models were tested to see which one describes better the dN and dS values as a function of the genetic distance. However, the models evaluated possess an important feature and this is their ability to show an accelerating or a declining trend of the fitted variable. Especially the cubic model incorporates both trends and can also reach a plateau. In this sense, we consider that we have selected flexible enough models to identify any saturation trend that might be exhibited by the substitution values. One more issue we have to mention is that not all datasets are informative enough for any model to be fitted (e.g. dN values of *Lozekia deubeli COX1*).

Considering all the above, it can be safely concluded that in 14 datasets the dS values are showing a declining trend (Table S2 in File S1) as genetic distance increases. Among these datasets those of *Codringtonia COX1*, *Partula COX1*, *Codringtonia COX2*, *Euhadra CytB*, *Arion ND1*, *Euhadra ND1* and *Euhadra ND4L* are included. These datasets are part of those datasets for which the McDonald-Kreitman test (no FDR correction) provided multiple positive selection indications (Table 2). At the same time, the datasets showing a declining trend of dN as genetic distance increases are 12 (Table S3 in File S1). Out of these, only two (*Codringtonia COX1*, *Albinaria2 COX1*) provide indications of abrupt saturation trend change when distance increases. The remaining datasets show a marginal declining trend of the dN values with elevating genetic distance. Consequently, dS values at high sequence divergence levels appear to be more saturated than dN values. Therefore, when high sequence divergence leads to substitutional saturation, the false positive outcome of the McDonald-Kreitman test should be more or less anticipated. As evolutionary distance increases, the saturation effect is becoming more pronounced, and is affecting dS to a greater degree than dN. At large divergence values dS is more likely to be underestimated and this will cause the dN/dS value for between species to be larger than expected. In a McDonald-Kreitman test this will bias estimates towards positive selection (N.I. <1.0). The opposite effect should hold for negative selection (underestimating dS makes it harder to get an NI value >1.0). It makes sense that this bias is weaker than the positive selection bias. Therefore, caution is needed in the interpretation of the findings of the McDonald-Kreitman test in those datasets where sequence divergence is very high and an increase of dN and dS with genetic distance is not occurring. Moreover, the positive relationship (results not shown) between tree length (Table 2) and positive MacDonald-Kreitman tests (no FDR correction) is also in favor of significant bias existing in this analysis. This bias results from saturation of synonymous substitutions as has already been claimed in other studies [61], [65], [66], [67], [68] but has not been explicitly shown.

### Maximum Likelihood Approach for Positive Selection

In the maximum likelihood approach for positive-selection-inference the results provide a different picture. The possibility of our findings being obscured by recombination has been eliminated since the recombination tests performed were negative. Therefore, based on the results of the M0 vs. M3 model comparison (Table 2), it is obvious that regardless of the locus under investigation, adequate indications exist to safely support the view that the selective pressure exerted along an mtDNA amino acid sequence is varying. Considering that the M0 model is actually an unrealistic one, its rejection is anticipated. Therefore, caution should be exerted when only M0 vs. M3 model indicate positive selection [78]. At the same time, there is not even a single dataset where the M1a vs. M2a model provides indications of positive selection. Consequently, according to this quite conservative test [78], the amino acids of the coding mtDNA sequences of pulmonates evolve under the strict control of purifying selection.

However, in the M7 vs. M8 model comparison (Table 2), some datasets do indicate that the sequence divergence of certain mtDNA genes is affected by positive selection. The datasets that such signs exist are *Partula*, *Placostylus*, *Sericata* and *Xerocrassa* in *COX1* and *Partula* in *Cytb*. Moreover, two of the datasets (*Partula*, *Xerocrassa*) pointed by the M7/M8 model comparison as exhibiting dN/dS >1 are also identified by the McDonald-Kreitman test (no FDR correction). One issue to consider regarding these cases where positive selection based on the M7/M8 LRT is identified is that no amino acids are recognized by the BEB method as being affected by positive selection. The M7/M8 model test is a quite stringent test of positive selection [78] and as such indications of positive selection based on this test should be given serious consideration. The fact that the BEB method cannot pinpoint the amino acids being positively selected is a common result in these analyses. It is probably due to the limited information included in the dataset analyzed to infer the sites. It is easier to decide whether such sites exist than to decide where they are. At this point it should be noted that the results of the present study are validating the findings of Goodacre et al. [6] that performed a maximum likelihood analysis on the *Cepaea nemoralis ATP8* gene and identified some sites as having a dN/dS value above 1. In the present study the same amino acids were identified via the NEB analysis of the M3 model.

Finally, in the dataset of *Albinaria ATP8* several amino acids were also found (NEB analysis, M3) to be exhibiting ω values greater than one. Nevertheless, it has been stated [79] and simulations studies have shown [78], that the power and accuracy of both the LRT and the BEB analysis are affected by the number and length of the sequences analyzed and the strength of positive selection [78], [79], [80]. For the LRT it has been found that longer sequences exhibit an increased probability of detecting adaptive evolution. At the same time very similar sequences carry little information, causing low power of the LRT [78]. On the other hand, multiple substitutions at the same site, as is the case in highly divergent sequences, result in the loss of information and reduced power in identifying positive selection. The simulation studies [78], [80] consider a tree length (a metric of sequence divergence) of 0.1 to 1 indicative of low divergence, 1.1 to 10 indicative of medium divergence, whereas values above 10 are considered to be of high divergence. In the present study there is only one case (*Wainuia*) where the tree length (Table 2) fits within the low divergence range among the datasets that have shown some signs of positive selection based on the maximum likelihood approaches (Table 2). The remaining datasets are exhibiting medium to high sequence divergence with the majority of them (11/23) leaning towards medium. Regarding the number of sequences involved in our analyses, in all the datasets the sequences used are well above the limits set in the simulation studies [78], [80]. Finally, the length of the coding sequences analyzed in the datasets reported in Table 2, is well above the 100 amino acids minimum length required [79], [80] for these analyses. Exceptions to this are the *ATP8* gene datasets of *Albinaria* and *Cepaea nemoralis* (Table 1). Therefore, for the datasets of pulmonates that have shown signs of positive selection based on the M7/M8 LRT, there is no reason to question this finding (Table 2). At this point we have to note that *ATP8* is a gene consistently identified in other animal groups as evolving (even after correction for multiple substitutions, [10], [13]) under the effect of positive selection [10], [13], [15]. In the datasets of *ATP8* of *Albinaria* and *Cepaea* the NEB analysis identified specific sites as exhibiting values of dN/dS >1. A*TP8* could be considered as evolving under the effect of positive selection, however we argue that it is not since: a) it is only the NEB analysis indicating positive selection and b) the length of this particular gene is below the 100 amino acids limit set by simulation studies.

Summarizing the findings from the mtDNA genes, a significantly high number (4/37 or 10.8%) of land-snail genera exhibit signs of positive selection (Table 2, M7/M8 model comparison) in *COX1* gene and one out of two in *Cytb*. In the remaining mtDNA genes no signs of positive selection were detected, however it has to be noted that the number of land-snail genera investigated in all remaining mtDNA genes was significantly smaller than those approached in *COX1*. There was a single land-snail genus (*Partula*) in which both *COX1* and *Cytb* were found to be evolving under the effect of diversifying selection.

Two recent examples of possible adaptive evolution of mtDNA were associated with dramatic metabolic differences in the habits of snakes and bats [10], [15]. In these studies the branch-site models test [81] investigating for episodic positive selection, was implemented. In pulmonates, it is possible that the patterns of aestivation in summer and hibernation in winter reflect changes in oxygen utilization that might drive strong diversification. However, these traits are not sufficiently lineage-specific that they can account for the lineage specific patterns of mtDNA variation, so it is difficult to associate the pulmonate mtDNA evolution to specific life history traits.

### Indications of Selection in Cyc

In *Cyc* the McDonald-Kreitman test did not indicate any signs of positive selection in either of the datasets analyzed (Table 2). On the other hand, the maximum likelihood approach was in favor of positive selection shaping the diversity of this gene in *Albinaria*, and there was a consensus among all the pairwise model comparisons (Table 2). Furthermore, the BEB analysis in all model comparisons of *Albinaria Cyc*, indicated the presence of a single amino acid as exhibiting an ω value greater than 1 with a probability >0.99. In *Codringtonia Cyc* the LRTs did not reveal any signs of positive selection, however the BEB analysis identified a single amino acid (probability >0.99) as having a dN/dS value that exceeds 1. Although the LRT test of M7 versus M8 in *Codringtonia Cyc* (Table 2) was not statistically significant (p_value_ = 0.092), it was in favor of positive selection acting on the gene.

Given the close association between nuclear-encoded and mitochondrial-encoded proteins of the electron transport system (ETS) and the crucial role of the ETS in energy production, the co-adaptation between nuclear-encoded and mitochondrial-encoded proteins has been strongly selected during the evolutionary history of these complexes [45]. According to Willett and Burton [45] two different scenarios of selection/drift could generate intergenomic coevolution: (1) Selection pressure on ETS enzymes could be generated by the adaptation of a species (or population) to its environment leading to changes in both nuclear-encoded and mitochondrial-encoded subunits of ETS complexes. (2) High mutation rates in mtDNA, especially when coupled with fluctuating population sizes, may lead to fixation of deleterious amino acid substitutions and provide selection pressure for subsequent compensatory changes in nuclear genes, a two-step process that could be the driving force of coevolution in ETS enzymes. One of the nuclear-encoded ETS genes that has repeatedly been found to be consistent with mtDNA/nuclear coevolution is the Cytochrome c (*Cyc*) gene [43], [45], [82]. The signs of selection, identified by the maximum likelihood approach (Table 2), in the *Cyc* gene of *Albinaria* and *Codringtonia* lend slim support to the cyto-nuclear coevolution hypothesis analyzed above, since it is based upon a single adaptive amino acid substitution. The amplification of the whole *Cyc* gene in the two studied genera, and the inclusion of additional land-snail genera could shed more light on this intriguing finding.

### Cyto-nuclear Coevolution

Sequence polymorphism surveys among *Tigriopus californicus* populations show evidence for positive selection at *Cyc* but negative selection at the interacting mitochondrial genes [45]. The same pattern is revealed here in *Cyc* of *Albinaria* and *Codringtonia* and one of its mtDNA-encoded counterparts, *COX1*. This pattern, as was the case in *Tigriopus californicus*, is consistent with the model of asymmetric compensatory coevolution discussed in Rand et al. [42]. At this point we have to note that the selection pattern of *Cyc* in land-snails is similar to that of *Tigriopus*, however it is not recorded between conspecific populations of land-snails. Instead, it is occurring between congeneric species in both land-snail genera that were assessed for *Cyc* polymorphism. However, one cannot rule out the possibility of this pattern being evident between conspecific land-snail populations as well. Such an investigation was not performed in the present study. The smaller spatial or taxonomical level that positive selection can be detected is an issue that needs to be further explored.

It is important to note here that the two land-snail genera, *Albinaria* and *Codringtonia*, are in the two extreme ends of land-snails’ diversity. *Albinaria* is a highly speciose genus with more than 200 taxa distributed in Greece [83], whereas *Codringtonia* is an endemic land-snail genus with six species distributed in mainland Greece [84]. At the same time premating isolation mechanisms are in effect non-existent in *Albinaria*, thus hybrids are very frequent [85]. On the other hand, hybridization has not been reported in *Codringtonia*. The fact that positive selection acting on a nuclear-encoded gene *Cyc* that interacts with mtDNA encoded counterparts is primarily detected in *Albinaria*, is in favor of the hypothesis of cyto-nuclear incompatibility acting as a post-mating isolation mechanism in *Albinaria*. This in turn could mean that the interactions between nuclear and mitochondrial genomes represent an important component of hybrid breakdown and species formation in land-snails as has already been suggested for other invertebrates such as *Tigriopus californicus* [86].

### Conclusions

According to the phylogenetic and population genetic analyses performed on the mtDNA sequences datasets, it can be said that the signals for positive selection obtained from the two different kinds of analyses are partially congruent. The McDonald-Kreitman test provided several indications of positive selection acting on the mtDNA genes evaluated. However, a thorough analysis of the substitution patterns exhibited by the mitochondrial datasets, and the identification of a strong positive relationship between tree length and positive MacDonald-Kreitman tests (no FDR correction), revealed that this test is biased towards identifying positive selection when sequence divergence is high and mutational saturation is inevitable. At the same time, the phylogenetic inference of positive selection refuted most of the McDonald-Kreitman tests findings and identified four datasets of *COX1* and one of *Cytb* as showing signs of positive selection. In the nuclear gene datasets McDonald-Kreitman tests did not produce any significant results, whereas the phylogenetic methods indicated that in both land snail genera approached, signs of positive selection are present. Overall, our findings indicate that: 1) slim support for the cyto-nuclear co-evolution is present, 2) the elevated rates of mtDNA polymorphims and divergence in pulmonates do not appear to be due to pervasive positive selection, 3) more stringent tests show that spurious positive selection is uncovered when distant taxa are compared and 4) there are significant examples of positive selection acting in some cases, so it appears that mtDNA evolution in pulmonates can escape from strict deleterious evolution suggested by the Muller’ s ratchet effect. The strict prediction of accumulation of deleterious mutations in non-recombining genomes is not upheld in land-snails, given a few cases of positive selection detected. It may be that mtDNA experiences occasional recombination permitting episodes of positive selection, or that occasional beneficial mutations can out-weigh the deleterious consequences of all linked polymorphisms combined. Resolution of this issue requires mechanistic studies that are beyond the scope of this report. Nevertheless, it can be claimed that negative selection does not seem to be an obligatory signature of the mtDNA evolution of pulmonates.

## Supporting Information

File S1 **Supporting figures and tables.** Figure S1. Mean levels of sequence divergence between species (mean inter-species divergence) and within species (mean intra-species divergence) based on the Kimura-2p genetic distance model. The+symbol denotes genera with data limitations that did not permit the calculation of a valid estimate, whereas++denotes genera for which only sequences of a single species were available. Figure S2a. The dS values as a function of the genetic distance using three different models. The best fitted model is in bold line. Blue color corresponds to the linear model, red to the quadratic and green to the cubic model. Figure S2b. The dS values as a function of the genetic distance using three different models. The best fitted model is in bold line. Blue color corresponds to the linear model, red to the quadratic and green to the cubic model. Figure S2c. The dS values as a function of the genetic distance using three different models. The best fitted model is in bold line. Blue color corresponds to the linear model, red to the quadratic and green to the cubic model. Figure S3a. The dN values as a function of the genetic distance using three different models. The best fitted model is in bold line. Blue color corresponds to the linear model, red to the quadratic and green to the cubic model. Figure S3b. The dN values as a function of the genetic distance using three different models. The best fitted model is in bold line. Blue color corresponds to the linear model, red to the quadratic and green to the cubic model. Figure S3c. The dN values as a function of the genetic distance using three different models. The best fitted model is in bold line. Blue color corresponds to the linear model, red to the quadratic and green to the cubic model.
(PDF)
