# Correction: Animal Mitochondria, Positive Selection and Cyto-Nuclear Coevolution: Insights from Pulmonates

**DOI:** 10.1371/annotation/f4fbb8ac-a185-4589-8c0a-28668283bc63

**Published:** 2013-10-15

**Authors:** Aristeidis Parmakelis, Panayiota Kotsakiozi, David Rand

There was an error in the list of accession numbers in Supplementary File S1 in the Supporting Information. Please view the correct version of S1 here: https://dl.dropboxusercontent.com/u/63557304/File_S1_Updated.pdf

