# Supplementary material for: Animal Mitochondria, Positive Selection and Cyto-Nuclear Coevolution: Insights from Pulmonates
Source: PLoS One. 2013 Apr 19;8(4):e61970. doi: 10.1371/journal.pone.0061970 (PMC3631144; doi:10.1371/journal.pone.0061970)
Supplement: File S1 — Supporting figures and tables. Figure S1. Mean levels of sequence divergence between species (mean inter-species divergence) and within species (mean intra-species divergence) based on the Kimura-2p genetic distance model. The+symbol denotes genera with data limitations that did not permit the calculation of a valid estimate, whereas++denotes genera for which only sequences of a single species were available. Figure S2a. The dS values as a function of the genetic distance using three different models. The best fitted model is in bold line. Blue color corresponds to the linear model, red to the quadratic and green to the cubic model. Figure S2b. The dS values as a function of the genetic distance using three different models. The best fitted model is in bold line. Blue color corresponds to the linear model, red to the quadratic and green to the cubic model. Figure S2c. The dS values as a function of the genetic distance using three different models. The best fitted model is in bold line. Blue color corresponds to the linear model, red to the quadratic and green to the cubic model. Figure S3a. The dN values as a function of the genetic distance using three different models. The best fitted model is in bold line. Blue color corresponds to the linear model, red to the quadratic and green to the cubic model. Figure S3b. The dN values as a function of the genetic distance using three different models. The best fitted model is in bold line. Blue color corresponds to the linear model, red to the quadratic and green to the cubic model. Figure S3c. The dN values as a function of the genetic distance using three different models. The best fitted model is in bold line. Blue color corresponds to the linear model, red to the quadratic and green to the cubic model. (PDF) [file pone.0061970.s001.pdf]

Supporting Information File of the original article entitled:

ANIMAL MITOCHONDRIA, POSITIVE SELECTION AND CYTO-NUCLEAR COEVOLUTION: INSIGHTS FROM PULMONATES

Aristeidis Parmakelis[1,*], Panayiota Kotsakiozi[2], David Rand[3]

[1] Department of Ecology and Taxonomy, Faculty of Biology, National and Kapodistrian University of Athens, Athens, Greece.
[2] Department of Animal and Human Physiology, Faculty of Biology, National and Kapodistrian University of Athens, Athens, Greece.
[3] Department of Ecology and Evolutionary Biology, Brown University, Providence, RI 02912, USA.

[*]**Corresponding author**: Aristeidis Parmakelis, Department of Ecology and Taxonomy, Faculty of Biology, National and Kapodistrian University of Athens, Panepistimioupoli Zografou, GR-15784, Athens, Greece. **Tel.:** ++30 210 7274736, **Fax:** ++30 210 7274736, **E-mail:** aparmakel@biol.uoa.gr.

Running title: mtDNA selection and cyto-nuclear coevolution

Keywords: Hybrid breakdown; land-snails; mtDNA; natural selection

Materials and Methods

mtDNA sequence data retrieved from GenBank

The mtDNA genes represented in the sequences retrieved from GenBank were (*COX1*) *Cytochrome Oxidase Subunit 1* (76.8%), (*COX2*) *Cytochrome Oxidase Subunit 2* (9%), (*Cytb*) *Cytochrome b* (4.3%), (*ND1*) *NADH Dehydrogenase Subunit 1* (4.4%), (ND4L) *NADH Dehydrogenase Subunit 4L* (2.7%) and (*ATP8*) *ATPase Subunit 8* (2.8%).

DNA extraction protocols, PCR amplification conditions and sequencing of mtDNA gene fragments generated for this study

DNA extraction and amplification methods applied in *Codringtonia* specimens to generate the *COX1* fragment, are provided in [1]. In total we generated the *COX1* sequences of five *Codringtonia* specimens (Table S1). The specimens of *Agathylla*, *Albinaria* used to generate mtDNA sequence data for this study were preserved either in absolute or 70% ethanol solution. Total genomic DNA was isolated from the foot muscle of the specimens. To overcome problems of polymerase chain reaction (PCR) inhibition by mucopolysaccharides, DNA was extracted using the CTAB 2x (hexadecyl-trimethyl-ammonium bromide) protocol of [2] as described in [3]. DNA was ultimately extracted from 29 adult *Agathylla* and 40 *Albinaria* individuals (Table S1). Specimens were assigned to species based on features of their shells and/or genital anatomy. Detailed information on the origin of the specimens used in this study is provided in Table S1. Two mtDNA markers were amplified in *Agathylla* specimens, the cytochrome oxidase subunit I (*COX1*) and the cytochrome oxidase subunit II (*COX2*). In *Albinaria* specimens only the *COX1* marker was amplified. For the amplification of the *COX1* gene fragment either the primer pair LCO1490/HCO2198 [4] or the primer pair C1-J-1718/C1-N-2191 [5] were used. For the amplification of the *COX2* gene, we used the primers reported in Hugall *et al*. [6]. The fragments targeted for *COX1* and *COX2* were 708 and 580 bp, respectively. Each PCR was performed in a 50 uL volume, where 1-2 uL of template DNA was mixed with 0.2 mM dNTPs, 0.4 mM of each primer, and 1 unit of Taq Polymerase. The concentration of the $MgCl_2$ was 3.5 mM. Thermocycling was performed in either a MyCycler (Biorad) or a TProfessional (Biometra) thermocycler. The cycle programs in all three mtDNA genes comprised an initial denaturation step at 95 °C for 3 min, followed by 40 cycles of 15 sec at 95 °C, 1 min at 45 °C, 1.5 min at 72 °C. The cycling was ended with 10 min sequence extension at 72 °C. Automated sequencing of both strands of each mtDNA fragment was performed in a PE-ABI3740 automated sequencer (using Big-Dye terminator chemistry). The primers in the sequencing reactions were the same as in the PCR amplifications. Accession numbers of sequences are reported in Table S1.

Land-snail genera in which the Cyc gene was amplified and analyzed

Both *Codringtonia* and *Albinaria* are rock-dwellers and are found in Greece. *Codringtonia* is an endemic pulmonate genus of Greece maintaining six species in the region [1]. *Albinaria* is distributed around the north-eastern coasts of the Mediterranean, exhibiting a high degree of morphological differentiation, especially in southern Greece and in the external insular Hellenic arc. More than 200 taxa (species and subspecies) of *Albinaria* have been described [7].

Nucleic acids extraction, cDNA synthesis, RT-PCR, and cloning of the Cyc amplicons

Land-snails specimens were collected from the field and preserved alive until further process. All specimens were treated according to relevant national and international guidelines. The foot muscle of each specimen was frozen and ground in liquid nitrogen. The total RNA was extracted from the tissue powder using the RNeasy Protect Mini Kit (Qiagen) and following the manufacturer's protocol. For the cDNA synthesis the M-MLV Reverse Trancriptase (Invitrogen) was used following the instructions suggested in the respective Invitrogen protocol. The cDNA served as template in a PCR aiming to amplify a fragment of the *Cyc* gene. All PCRs were conducted on a Biometra TProfessional thermal cycler. Several primer pairs designed based on *Cyc* sequences of other invertebrate species available in GenBank, were tested in the amplification procedure. The primers that were successful in amplifying the targeted gene in the two land-snail genera of concern are available upon request from the authors. Following the PCR amplification, the amplicons that were of the expected size (visually verified through agaroze gel electrophoresis) were cloned using the TOPO-TA cloning kit (Invitrogen). From each individual, five to eight transformed colonies were selected, and the size of the DNA insert was screened by PCR using the M13F/M13R primer pair of the pCRII-TOPO vector. In most of the cases the correct size insert was obtained, and was subsequently sequenced in both directions. Produced *Cyc* chromatograms were visually inspected and edited using CodonCode Aligner v. 2.0.6. The accession numbers of *Cyc* sequences produced are given in Table S1.

Sequences alignment and phylogenetic analyses

All sequences sets were aligned using CodonCode Aligner v. 2.0.6 and the inferred alignment was manually inspected for spurious gaps. Unrooted phylogenetic trees were constructed with MrBayes 3.1.2 [8], using either partitioned or non-partitioned data depending on the dataset. If the dataset was partitioned, a substitution model was separately fitted to the first, second, and third codon position of the analyzed locus. MrMTgui (http://www.softpedia.com/get/Science-CAD/MrMTgui.shtml), the cross platform interface for Modeltest [9], was used in order to select the substitution models to implement in the Bayesian analysis. The model selection criterion was that of the (AIC) Akaike Information Criterion [10]. In several datasets the number of sequences involved were more than 150 and some of them were identical to each other as judged after their pair-wise comparisons performed using MEGA v.4 [11] and implementing the Kimura two-parameter model [12]. Aiming at reducing the computation time of the Bayesian analysis, only the divergent haplotypes of each dataset were retained in the analysis. There was a dataset (*Praticolella*: Perez K.E., unpublished *COX1* data) that did not produce an adequately resolved Bayesian tree and thus was excluded from the analysis.

In order to select the appropriate outgroup for each phylogenetic analysis we followed two different approaches. In the case of published datasets we used the same outgroup as the one suggested by the authors and in some cases we used additional ones. In the case of unpublished data we performed a BLAST (http://blast.ncbi.nlm.nih.gov/Blast.cgi) search to retrieve an appropriate outgroup from GenBank , as well as searched the literature for a taxon related to the genus under question, in order to find some other suggestions of possible outgroups based on non-molecular data. In the latter case, if the respective mtDNA sequences of the suggested outgroup existed in GenBank , it was used in the phylogenetic analysis. In any other case the phylogenetic analysis was performed without an outgroup taxon.

Inferring selection using maximum likelihood methods, models' details and the likelihood ratio test

Model M1a is a neutral model which divides the codon sites into two categories, one having conserved sites with $\omega_0 = 0$ and the other involving neutral sites with $\omega_1 = 1$. Model M2a allows an additional category of sites with ω_2 estimated from the data, thus accommodating positively selected sites. Models M7 and M8 assume that ω follows a beta distribution with the shape parameters estimated in the interval (0, 1), and M8 includes one additional category to account for positively selected sites [13]. In all these models the rate of synonymous substitutions (dS) is constant among sites, while the rate of non-synonymous substitutions (dN)

is variable [14]. Following the suggestions of the PAML software [15] the site models pairs that appear to be particularly useful for real data analysis, are the M1a versus M2a and M7 versus M8. However, we also compared model M0 versus M3 in order to see if the selective pressure is uniform among sites. The strength of support of positive selection was calculated by comparing twice the log-likelihood difference in a chi-square test (Likelihood Ratio test, LRT) with four (M0 versus M3) or two (M1a versus M2a, and M8 versus M7) degrees of freedom. Aiming to reduce the computation time in all the calculations of the log-likelihood of each tree under the M1a, M2a, M3, M7 and M8 models, the initial branch lengths were those estimated under the M0 model, as suggested in PAML's manual. All models can suffer from localized optima and therefore were run (including M0) several times until the log-likelihood values between different runs converged.

Testing the datasets for recombination

All the tests for selection that are based on phylogeny inference, assume that there has been no recombination within any of the sequences analyzed. The heteroplasmy hypothesis, that could provide the basis for recombination [16] has been rejected in pulmonates [17,18]. Furthermore, according to Galtier et al. [19] the peculiarities of the mitochondrial mutation process can generate recombination-like patterns of sequence variation. The same authors conclude that because of the lack of power of recombination detection methods, the prevalence of mitochondrial recombination across animals is currently difficult to assess. Nevertheless, all the mtDNA datasets in which multiple sequences from a single population were present, were evaluated for the presence of recombination. Recombination was assessed using the RDP3 software [20] following the guidelines of the software' s manual and the suggestions provided in textbooks [21].

Investigating the substitutions' saturation through model fitting

In order to investigate the issue of substitution saturation argument we estimated (in all datasets) the pairwise estimates [22] of dN and dS and plotted them against the respective pairwise genetic distance that was estimated based on the Jukes-Cantor model [23]. Relationships between the genetic distance and dN and dS were modeled separately using the ordinary least squares method. The cubic, quadratic and the linear function of genetic distance were tested in order to account for curvilinear relationships. The curvilinear model (quadratic or cubic) was selected if (1) the linear and the high-order term were significant and (2) the polynomial (i.e. quadratic or cubic) model was more informative than the linear one. To avoid over-fit due to over-parameterization, the additional information provided by the polynomial models was assessed by the change in information according to the Bayesian Information Criterion (BIC) [24]. The AIC [10], was also used (results not shown) but we selected to present the results of BIC because it is more strict in model selection, and is placing a higher penalty value when a model includes many parameters [24,25]. Furthermore, BIC performs better when large datasets are involved in the analyses [24]. BIC weights (w_i), normalized across the three models tested to sum to one, were derived to evaluate the relative likelihood of each of the three fitting models. A model was considered to be relevant when its partial weighting was at least 5% greater than that of the linear model [25,26]. All model fitting analyses were performed in the R-environment [27].

Results

Authenticity of the Cyc sequences generated

The authenticity of the *Cyc* sequences was verified using a BLAST search. All produced sequences were homologous to published *Cyc* sequences of other invertebrate and vertebrate species. Furthermore, the translation of the sequences into their protein product returned an amino acid sequence that was continuous with no spurious stop codons and exhibited a high level (above 73%) of homology with published nuclear cytochrome c proteins.

Levels of sequence divergence between and within species of pulmonates

Levels of sequence divergence are presented in Figure S1. It can be seen that the pulmonate genus *Candidula* exhibits levels of mean *COX1* sequence divergence between species that are close to 25%. In *COX2*, *Agathylla* maintains a mean value of divergence as high as 19%. The mean divergence between species in *Cytb* is elevated to 17% in *Euhadra*. For the remaining mtDNA genes, the sequences of less than three pulmonate genera are available for comparison. Nevertheless, it is quite impressive that the mean level of divergence between different *Arion* species is 35% in the *ND1* locus. The respective levels of *ND1* in

Euhadra are also quite high mounting up to almost 21%. At the same time, different species of *Euhadra* diverge as much as 28% in *ND4L*. Based on *Euhadra*, the single pulmonate genus for which the sequences of four mtDNA genes are available, it seems that within a specific genus among *COX1*, *Cytb*, *ND1* and *ND4L*, the gene that exhibits the highest mean levels of species divergence is *ND4L* and *ND1* follows immediately after, then *Cytb* and finally *COX1*. Among the remaining pulmonate genera for which sufficient data exist for at least two of these genes, the above mentioned pattern observed in *Euhadra* seems to be true for *Arion*, *Partula* and marginally for *Pupilla* as well.

In *COX1 Euhadra* exhibits the highest levels that reach up to 9.8%. In *COX2* different populations of *Agathylla* diverge as much as 13.8%. The mean within species divergence of *Partula* is 7.7% in *Cytb*. In *Cepaea nemoralis* the mean sequence divergence between two populations is 12% in *ATP8*. Finally, conspecific populations of *Euhadra* exhibit a mean divergence level of 9.7% in *ND1*.

Table S1. Details on the geographic origin and species status of *Agathylla*, *Albinaria* (*Albinaria2* of Table 1) and *Codringtonia* specimens used in the study. The accession numbers of the generated sequences are provided for each locus and genus. For *Codringtonia* the *Cyc* accession numbers are given, and only those *COX1* accession numbers of *Codringtonia* specimens that are not already published [1]. For *Albinaria* we have generated 40 *COX1* sequences and 19 *Cyc* sequences.

Sample code	*COX1* Accession Numbers	*COX2* Accession Numbers	*Cyc* Accession Numbers	Species Name	Country of origin	Locality
***Agathylla* specimens**						
Aa84742	KC756068	KC756096		*Agathylla abrupta*	HUNGARY	Peljesac-fsz., Pijavicino K 2 km, emlékmű
Ab86066	KC756069	KC756097		*Agathylla biloba biloba*	ALBANIA	Periferi Shkodër, Shkodër, SE side of the castle hill (40 m) [limestone rocks]
Ab95491	KC756070	KC756098		*Agathylla biloba ssp.*	ALBANIA	Periferi Elbasan, 1.5 km S of Petrësh (= S of Graçen), along the Tiranë - Elbasan main road (500 m) [limestone rocks]
Ab95492	KC756071	KC756099		*Agathylla biloba ssp.*	ALBANIA	Periferi Berat, Qafa e Gllavës, along the Berat - Këlcyrë road (900 m) [limestone rocks]
Abd86065	KC756072	KC756100		*Agathylla biloba dabovici*	ALBANIA	Periferi Shkodër, Laç-Qyrsaç (20 km SE of Shkodër) left side of the dam of Liqeni i Vaut të Dejës (55 m) [rocks, in a spring]
Abd97076	KC756073	KC756101		*Agathylla biloba dabovici*	ALBANIA	Periferi Shodër, "Shenmarki" ca. 1.2 km NE of Mjedë, near river Drin (42.01468+19.62839)
Abm85893	KC756074	KC756102		*Agathylla biloba martae*	ALBANIA	Periferi Tiranë, Qafa e Fangul, 9 km E of Ibë towards Kllojkë, over the N side of the gorge of the Pr. i Murdharit (700 m)
Ae84822	KC756075	KC756103		*Agathylla exarata*	BOSNIA AND HERZEGOVINA	Velež hg., Podvelež, D. Gnojnice K 3 km
Ae84823	KC756076	KC756104		*Agathylla exarata*	BOSNIA AND HERZEGOVINA	Neretva-vgy., Počitelj
Ae84829	KC756077	KC756105		*Agathylla exarata*	HUNGARY	Klek ÉNY 8 km (Neumnál) a rabai elágtól É-ra
Ae84830	KC756078	KC756106		*Agathylla exarata*	HUNGARY	Lovorje (Neumnál)
Ae97067	KC756079	KC756107		*Agathylla exarata*	HUNGARY	Baćina, 2 km on the road towards Ploče, S shore of Baćinsko Jezero, 15 m [limestone rocks and macchia vegetation]

Af84754	KC756080	KC756108		*Agathylla formosa*	HUNGARY	Komolac temetődomb (Dubrovniknál)
Ag97071	KC756081	KC756109		*Agathylla goldi*	MONT	Lovćen Mts, gorge ca. 8 km from Kotor on the Kotor–Njeguši road, 400 m [limestone rocks, macchia vegetation]
Agh97068	KC756082	KC756110		*Agathylla goldi herminiana*	MONTENEGRO	Lovćen Mts, ca. 16 km from Kotor on the Kotor–Njeguši serpentine road, curve no. 23, 809 m [limestone rocks]
Al84751	KC756083	KC756111		*Agathylla lamellosa*	HUNGARY	Čavtat
Al97075	KC756084	KC756112		*Agathylla lamellosa*	MONTENEGRO	Čanj, near Petrovac
Alat84748	KC756085	KC756113		*Agathylla strigillata latestriata*	HUNGARY	Peljesac-fsz., Pijavicino K 2 km, emlékmű
Am2010/53	KC756086	KC756114		*Agathylla merditana*	ALBANIA	Shkoder district, Prokletije Mts, Nicaj-Shosh, rocky stream W (above) of the village 980m
An86067	KC756087	KC756115		*Agathylla neutra*	ALBANIA	Periferi Skrapar, 4 km SE of Çorovodë towards Zogas, by the right side of the canyon of Lumi i Osumit (400 m) [limestone rocks]
An95037	KC756088	KC756116		*Agathylla neutra*	ALBANIA	Periferi Skrapar, Mali i Tomorrit, 4.8 km NE of Çorovodë towards Radesh, over the gorge of Pr. i Çorovodës (475 m)
An97102	KC756089	KC756117		*Agathylla neutra*	ALBANIA	Periferi Shkodër, ca. 1.5 km upstream from dam at Koman, Liqeni i Komanit, left bank (180 m) [limestone rocks]
Anm95960	KC756090	KC756118		*Agathylla neutra merditana*	ALBANIA	Periferi Mat, in the gorge of Lumi i Matit, (Burrel - Milot), 11 km W of the conjunction to Ulëz (100 m)
Anm97072	KC756091	KC756119		*Agathylla neutra merditana*	ALBANIA	Periferi Pukë, 3 km N of Miliskë (16 km S of Fierzë) (567 m)
As83431	KC756092	KC756120		*Agathylla sulcosa*	HUNGARY	Veli Zaton (near Dubrovnik)
As97069	KC756093	KC756121		*Agathylla sulcosa*	HUNGARY	Trsteno SE 1 km along the road to Dubrovnik, 93 m [limestone rocks, roadside vegetation]
As97070	KC756094	KC756122		*Agathylla sulcosa*	HUNGARY	Slano, along the road towards Dubrovnik, 9 m [limestone rocks and roadside ruderalia]
Asa83441	KC756095	KC756123		*Agathylla sulcosa acicula*	HUNGARY	Komolac temetődomb (Dubrovniknál)

Ast84788	KC756068	KC756124		*Agathylla strigillata*	HUNGARY	Peljesac-fsz., Ston várfal
Albinaria specimens						
AaDia1	KC756125		End of this file	*Albinaria arcadica*	GREECE	Peloponnese, Diakopto gorge
AaDia2	KC756126		End of this file	*Albinaria arcadica*	GREECE	Peloponnese, Diakopto gorge
AaDia3	KC756127		End of this file	*Albinaria arcadica*	GREECE	Peloponnese, Diakopto gorge
AaDia4	KC756128		End of this file	*Albinaria arcadica*	GREECE	Peloponnese, Diakopto gorge
AaKast3	KC756129		End of this file	*Albinaria anatolica*	GREECE	Dodecanese, Kastelorizo
AaKast4	KC756130			*Albinaria anatolica*	GREECE	Dodecanese, Kastelorizo
Aarcad1	KC756131		KC756182	*Albinaria arcadica*	GREECE	Peloponnese, Diakopto gorge
Aarcad2	KC756132		KC756183	*Albinaria arcadica*	GREECE	Peloponnese, Diakopto gorge
AbHel3	KC756133		End of this file	*Albinaria broemei*	GREECE	Peloponnese, Helmos-501
Ac150_1	KC756134		End of this file	*Albinaria caerulea*	GREECE	East Aegean, Kos-point 150
Ac150_2	KC756135			*Albinaria caerulea*	GREECE	East Aegean, Kos-point 150
Ac150_3	KC756136			*Albinaria caerulea*	GREECE	East Aegean, Kos-point 150
Ac150_4	KC756137			*Albinaria caerulea*	GREECE	East Aegean, Kos-point 150
Ac358_1	KC756138		End of this file	*Albinaria caerulea*	GREECE	Cyclades, Anafi-Monastiri-1-358
Ac358_2	KC756139			*Albinaria caerulea*	GREECE	Cyclades, Anafi-Monastiri-1-358
Ac358_3	KC756140			*Albinaria caerulea*	GREECE	Cyclades, Anafi-Monastiri-1-358
Ac358_4	KC756141			*Albinaria caerulea*	GREECE	Cyclades, Anafi-Monastiri-1-358
Ac359_1	KC756142		End of this file	*Albinaria caerulea*	GREECE	Cyclades, Anafi-Monastiri-1-359
Ac359_2	KC756143			*Albinaria caerulea*	GREECE	Cyclades, Anafi-Monastiri-1-359

Ac359_3	KC756144			*Albinaria caerulea*	GREECE	Cyclades, Anafi-Monastiri-1-359
Ac359_4	KC756145			*Albinaria caerulea*	GREECE	Cyclades, Anafi-Monastiri-1-359
Ac360_1	KC756146		End of this file	*Albinaria caerulea*	GREECE	Cyclades, Anafi-Chora-360
Ac360_2	KC756147			*Albinaria caerulea*	GREECE	Cyclades, Anafi-Chora-360
Ac360_3	KC756148			*Albinaria caerulea*	GREECE	Cyclades, Anafi-Chora-360
Ac360_4	KC756149			*Albinaria caerulea*	GREECE	Cyclades, Anafi-Chora-360
AcKosCas3	KC756150		End of this file	*Albinaria caerulea*	GREECE	East Aegean, Kos-Castle
AcKosCas4	KC756151			*Albinaria caerulea*	GREECE	East Aegean, Kos-Castle
Agris1	KC756152		KC756184	*Albinaria grisea*	GREECE	Sterea Ellada, Imittos Mt., above the University of Athens
AhProu3	KC756153			*Albinaria hians*	GREECE	Sterea Ellada, Proussos
AhProu4	KC756154			*Albinaria hians*	GREECE	Sterea Ellada, Proussos
Al50_1	KC756155		End of this file	*Albinaria lerosiensis*	GREECE	East Aegean, Kos-point 150
Al50_2	KC756156			*Albinaria lerosiensis*	GREECE	East Aegean, Kos-point 150
Al50_3	KC756157			*Albinaria lerosiensis*	GREECE	East Aegean, Kos-point 150
Al50_4	KC756158			*Albinaria lerosiensis*	GREECE	East Aegean, Kos-point 150
AlKos3	KC756159			*Albinaria lerosiensis*	GREECE	East Aegean, Kos-Ancient Temple
AlKos4	KC756160			*Albinaria lerosiensis*	GREECE	East Aegean, Kos-Ancient Temple
ApKarp3	KC756161		End of this file	*Albinaria proteus*	GREECE	Dodecanese, Karpathos
Apraec1	KC756162		KC756185	*Albinaria praeclara*	GREECE	Crete, Giouchtas Mt., Archanes
AvPar3	KC756163		End of this file	*Albinaria voithii*	GREECE	Peloponnese, Parori-near Sparti
AvPar4	KC756164		End of this file	*Albinaria voithii*	GREECE	Peloponnese, Parori-near Sparti

Codringtonia specimens						
Cc_Mal1	[1]			*Codringtonia codringtonii*	GREECE	Peloponnese, Agios Petros to Malevi Monastery
Cc_Math1	[1]			*Codringtonia condringtonii*	GREECE	Peloponnese, Mathias 552m
Ce_AA7	[1]			*Codringtonia elisabethae*	GREECE	Peloponnese, Ahladokampos to Argos
Ce_ST6_2	[1]			*Codringtonia elisabethae*	GREECE	Peloponnese, Steno to Tripoli
Ce_ST6_3	[1]		End of this file	*Codringtonia elisabethae*	GREECE	Peloponnese, Steno to Tripoli
Ceu_Mist4	[1]		KC756174	*Codringtonia eucineta*	GREECE	Peloponnese, Mistras
Ceu_Mist5	[1]		End of this file	*Codringtonia eucineta*	GREECE	Peloponnese, Mistras
Ceu_PAN_3	[1]		KC756175	*Codringtonia eucineta*	GREECE	Peloponnese, Panahaiko mt 1800m
Cg_MelR4_2	[1]		KC756176	*Codringtonia gittenbergeri*	GREECE	Peloponnese, Oreini Meligou 670m
Cg_MelR5_1	[1]		End of this file	*Codringtonia gittenbergeri*	GREECE	Peloponnese, Oreini Meligou 670m
Ch_MEN21_R2_1	KC756166		KC756177	*Codringtonia helenae*	GREECE	Peloponnese, Menalo Mt. 1455m
Ch_MEN31_R3	KC756167		End of this file	*Codringtonia helenae*	GREECE	Peloponnese, Menalo Mt. 1633m
Ch_TM4_2	[1]		KC756178	*Codringtonia helenae*	GREECE	Peloponnese, Tripoli to Menalo Mt.
Ci_Dia1	[1]		KC756179	*Codringtonia intusplicata*	GREECE	Peloponnese, Diakopto gorge
Ci_Dia2	[1]		KC756180	*Codringtonia intusplicata*	GREECE	Peloponnese, Diakopto gorge
Ci_Dia3	KC756168		KC756181	*Codringtonia intusplicata*	GREECE	Peloponnese, Diakopto gorge

Ci_Dia4	KC756169		End of this file	*Codringtonia intusplicata*	GREECE	Peloponnese, Diakopto gorge
Ch_MEN19_R4_2	KC756165		End of this file	*Codringtonia helenae*	GREECE	Peloponnese, Menalo Mt. 1053m
Cp_The1	[1]		End of this file	*Codringtonia parnassia*	GREECE	Sterea Ellada, Thermopiles

Table S2. The Bayesian Information Criterion (BIC) values, its corresponding weight (w_i), and the characteristic features of each of the three different models used to fit the dS values as a function of the genetic distance (Jukes-Cantor model). All the R^2 values provided are adjusted to account for the number of pairwise comparisons, since unadjusted R^2 are biased. In the weights column the best model selected for each data set is indicated in bold. For the quadratic and cubic model the values of the last slope indicating the onset of the decline of the dS values, are indicated in bold in the slope' s column. The equation for computing BIC is -2Log(L)+kLog(N) where L is the maximized value of the likelihood estimated by the model, k is the number of parameters (including the estimated parameter), and N is the number of observed values. For the linear model k=3, for the quadratic k=4 and for the cubic k=5.

	**Linear Model**				**Quadratic model**					**Cubic model**					
COX1	**BIC**	w_i	**Adj. R^2**	**Slope**	**BIC**	w_i	**Adj. R^2**	**Slope 1**	**Slope 2**	**BIC**	w_i	**Adj. R^2**	**Slope 1**	**Slope 2**	**Slope 3**
Achatinella	-26229.679	0.000	0.955	4.121	-26230.833	0.000	0.955	4.241	-0.600	-26841.032	**1.000**	0.959	2.750	21.181	**-78.423**
Agathylla	823.509	**0.953**	0.360	7.424	829.660	0.044	0.358	7.492	-0.228	835.224	0.003	0.358	4.630	24.518	-57.635
Albinaria2	672.247	0.000	0.643	7.729	613.886	0.000	0.671	0.405	35.734	583.032	**1.000**	0.686	11.690	-99.790	413.430
Amborytida	-1954.036	0.000	0.950	6.191	-2157.485	**0.947**	0.961	3.827	12.933	-2151.720	0.053	0.961	4.114	8.717	15.673
Arianta	-3157.074	0.000	0.825	8.018	-4894.210	0.041	0.870	3.297	19.246	-4900.532	**0.959**	0.870	2.442	28.010	**-22.509**
Arion	3106.699	**0.938**	0.285	8.179	3112.187	0.060	0.286	6.739	4.113	3119.245	0.002	0.285	4.787	18.946	-30.285
Ashmunella	-1513.280	0.000	0.962	6.135	-1554.575	0.000	0.964	7.582	-6.580	-1570.042	**1.000**	0.964	12.260	-50.307	102.906
Bulimulus	-9965.298	0.000	0.963	6.146	-10235.984	0.000	0.965	4.956	6.498	-10325.051	**1.000**	0.966	3.461	24.277	**-57.010**
Candidula	732.646	0.309	0.324	7.867	734.088	0.150	0.331	11.475	-10.872	731.524	**0.541**	0.345	-6.349	124.388	**-261.160**
Carinigera	346.137	0.000	0.755	12.310	-285.526	0.000	0.934	-5.172	71.194	-472.992	**1.000**	0.956	13.435	-126.496	544.765
Codringtonia	-2544.002	0.000	0.869	7.631	-2678.454	0.000	0.875	6.039	7.899	-3245.227	**1.000**	0.894	-1.359	95.392	**-259.599**
Elona quimperiana	-1045.302	0.000	0.963	7.512	-1476.465	0.000	0.980	0.497	24.867	-2011.314	**1.000**	0.991	6.409	-41.515	166.137
Euchemotrema hubrichti	-203.379	0.017	0.968	5.321	-210.691	**0.664**	0.971	-0.213	28.513	-209.228	0.319	0.971	4.475	-28.205	171.756
Euhadra	-4763.159	0.000	0.899	8.969	-11079.837	**0.968**	0.943	0.294	39.484	-11072.994	0.032	0.943	0.540	37.145	6.212
Everettia	-885.161	0.000	0.784	8.129	-973.277	**0.909**	0.803	3.210	20.410	-968.664	0.091	0.803	1.557	37.806	-51.719

Iberus	7807.008	0.000	0.761	12.405	4452.437	0.000	0.843	-5.284	63.899	2267.387	**1.000**	0.881	23.532	-232.685	813.647
Kovacsia kovacsii	-829.498	0.000	0.981	6.340	-1598.795	0.000	0.999	3.376	19.509	-1783.425	**1.000**	1.000	5.321	-15.272	148.999
Lozekia deubeli	-429.533	0.000	0.952	6.579	-606.483	0.000	0.983	2.949	20.630	-622.843	**1.000**	0.985	0.357	59.903	**-151.146**
Marmorana	-153.197	**0.626**	0.848	7.131	-146.909	0.027	0.848	6.912	0.910	-152.019	0.347	0.851	3.603	35.548	-95.561
Natalina	593.970	0.000	0.741	9.319	41.763	0.000	0.804	-2.183	40.291	-87.417	**1.000**	0.817	9.684	-56.620	229.911
Oreohelix	-7997.839	0.000	0.970	5.513	-8020.176	0.000	0.970	4.933	3.276	-8178.126	**1.000**	0.971	3.051	33.710	**-112.375**
Partula	-13086.764	0.000	0.788	6.790	-13821.173	0.000	0.802	3.998	14.857	-14102.875	**1.000**	0.807	-0.241	64.812	**-162.011**
Partulina	-355.179	0.000	0.900	5.915	-413.487	**0.534**	0.911	3.349	8.180	-413.211	0.466	0.912	1.825	19.282	-21.602
Paryphanta busbyi	-878.498	**0.836**	0.993	4.258	-875.048	0.149	0.993	4.025	8.263	-870.485	0.015	0.993	3.916	18.802	-265.839
Placostylus	-221.651	0.180	0.896	5.371	-224.423	**0.719**	0.899	6.204	**-3.025**	-220.491	0.101	0.899	4.934	7.694	-22.174
Pupilla	-166.873	0.000	0.980	5.498	-182.707	0.011	0.986	4.120	7.336	-191.684	**0.989**	0.988	7.544	-36.695	144.105
Satsuma	2247.704	0.000	0.812	8.499	-1233.043	0.000	0.846	0.799	28.407	-1287.038	**1.000**	0.847	3.010	8.765	49.150
Sericata	67.417	0.000	0.832	9.059	-77.221	0.002	0.892	-1.939	46.674	-90.173	**0.998**	0.897	6.412	-33.952	213.159
Solatopupa	-75.691	0.000	0.894	7.103	-94.433	**0.862**	0.914	2.571	19.973	-90.763	0.138	0.914	4.177	2.502	51.348
Succinea	-1996.566	0.000	0.907	5.939	-2507.443	0.000	0.928	1.853	18.802	-2812.912	**1.000**	0.938	8.914	-49.559	176.564
Trochulus	3016.058	0.000	0.728	8.405	1921.821	**0.507**	0.785	-0.670	39.796	1921.875	0.493	0.785	1.206	20.721	50.826
Vertigo	-7491.648	0.000	0.974	6.284	-8756.511	0.000	0.984	4.512	8.209	-8867.641	**1.000**	0.985	5.604	-5.643	43.039
Wainuia	-961.070	0.000	0.976	5.934	-1156.175	**0.884**	0.988	3.106	18.181	-1152.110	0.116	0.988	3.609	9.930	35.908
Xerocrassa	1533.022	0.000	0.747	10.736	-3150.658	0.000	0.867	-5.166	63.954	-5275.137	**1.000**	0.900	15.323	-136.381	555.493
COX2															
Agathylla	697.850	**0.866**	0.251	4.988	701.751	0.123	0.253	7.814	-5.601	706.516	0.011	0.253	3.405	16.472	-31.777
Codringtonia	617.550	0.000	0.724	4.615	-9.077	0.000	0.772	7.769	-9.373	-2335.183	**1.000**	0.887	-3.303	75.011	**-156.379**

Gnarosophia bellendekerensis	-163.329	0.000	0.852	8.697	-829.697	0.000	0.929	1.463	27.468	-881.180	**1.000**	0.933	6.544	-25.030	134.593
Sphaerospira	984.040	0.000	0.781	9.777	-97.185	0.001	0.881	-1.502	40.407	-111.218	**0.999**	0.882	1.732	13.263	61.982
Cytb															
Euhadra	2374.881	0.000	0.584	7.751	2382.391	0.000	0.584	7.583	0.444	2254.781	**1.000**	0.611	-6.279	87.874	**-156.373**
Pupilla	-66.825	0.000	0.954	3.423	-114.712	0.036	0.989	6.165	-8.143	-121.310	**0.964**	0.991	8.250	-33.992	61.989
ND1															
Arion	729.488	0.000	0.372	2.383	663.374	0.000	0.425	5.667	-4.920	643.976	**1.000**	0.443	1.268	11.498	**-17.083**
Euhadra	-0.370	0.000	0.662	3.699	-124.474	0.000	0.683	5.885	-4.339	-266.557	**1.000**	0.705	0.690	19.791	**-31.439**
ND4L															
Euhadra	2370.802	0.000	0.479	4.719	2377.791	0.000	0.478	5.042	-0.661	2352.350	**1.000**	0.487	-1.637	31.947	**-44.953**
ATP8															
Albinaria	-1150.427	0.000	0.861	2.458	-1185.166	**0.949**	0.872	1.262	4.212	-1179.315	0.051	0.872	1.079	6.106	-4.997
Cepaea nemoralis	-1733.936	0.038	0.987	3.574	-1728.376	0.002	0.987	3.376	0.838	-1740.413	**0.960**	0.987	1.531	19.106	**-47.438**

Table S3. The Bayesian Information Criterion (BIC) values, its corresponding weight (w_i), and the characteristic features of each of the three different models used in our effort to fit the dN values as a function of the genetic distance (Jukes-Cantor model). All the R^2 values provided are adjusted to account for the number of pairwise comparisons, since unadjusted R^2 are biased. In the weights column the best model selected for each data set is indicated in bold. For the quadratic and cubic model the values of the last slope indicating the onset of the decline of the dN values, are indicated in bold in the slope' s column. The equation for computing BIC is -2Log(L)+kLog(N) where L is the maximized value of the likelihood estimated by the model, k is the number of parameters (including the estimated parameter), and N is the number of observed values. For the linear model k=3, for the quadratic k=4 and for the cubic k=5.

	**Linear Model**				**Quadratic model**					**Cubic model**					
COX1	**BIC**	***w_i***	**Adj. R^2**	**Slope**	**BIC**	***w_i***	**Adj. R^2**	**Slope 1**	**Slope 2**	**BIC**	***w_i***	**Adj. R^2**	**Slope 1**	**Slope 2**	**Slope 3**
Achatinella	-51200.459	0.000	0.812	0.351	-55420.697	0.000	0.893	-0.057	2.036	-56316.465	**1.000**	0.905	0.198	-1.693	13.426
Agathylla	-2807.818	0.000	0.421	0.253	-2874.727	**0.893**	0.495	-0.140	1.293	-2870.482	0.107	0.496	-0.283	2.515	-2.821
Albinaria2	-6643.877	0.000	0.520	0.055	-6661.883	0.000	0.535	0.015	0.189	-6681.979	**1.000**	0.550	-0.070	1.171	**-2.884**
Amborytida	-6023.912	0.000	0.682	0.174	-6839.032	0.000	0.883	-0.156	1.809	-6898.430	**1.000**	0.892	-0.021	-0.183	7.405
Arianta	-39148.702	0.000	0.660	0.233	-46716.463	**0.971**	0.904	-0.138	1.359	-46709.443	0.029	0.904	-0.130	1.283	0.182
Arion	-10407.190	0.000	0.371	0.349	-10538.441	**0.934**	0.411	-0.044	1.081	-10533.145	0.066	0.411	-0.182	2.109	-2.055
Ashmunella	-6206.572	0.000	0.798	0.251	-7438.112	0.308	0.939	-0.335	2.668	-7439.732	**0.692**	0.939	-0.504	4.249	**-3.720**
Bulimulus	-24777.835	0.000	0.471	0.158	-26685.323	0.000	0.683	-0.230	2.120	-27349.115	**1.000**	0.735	0.190	-2.873	16.010
Candidula	-2405.728	0.000	0.406	0.310	-2560.212	0.363	0.569	-0.268	1.596	-2561.339	**0.637**	0.575	0.115	-1.216	5.283
Carinigera	-4070.557	0.000	0.589	0.094	-4348.287	0.000	0.767	-0.060	0.603	-4367.697	**1.000**	0.779	0.059	-0.635	3.356
Codringtonia	-27320.217	0.000	0.676	0.110	-29294.194	0.000	0.819	-0.021	0.631	-30410.622	**1.000**	0.870	-0.209	2.787	**-6.181**
Elona quimperiana	-5858.670	0.000	0.979	0.324	-7181.741	0.000	0.997	0.014	1.097	-7471.416	**1.000**	0.998	-0.068	2.016	**-2.298**
Euchemotrema hubrichti	-707.483	**0.795**	0.896	0.260	-704.341	0.165	0.897	0.443	-0.942	-701.519	0.040	0.897	0.103	3.168	-12.445
Euhadra	-72594.503	0.000	0.507	0.143	-87819.940	0.000	0.872	-0.208	1.408	-92361.794	**1.000**	0.914	0.113	-1.099	5.154
Everettia	-6979.082	0.000	0.440	0.199	-7188.015	0.000	0.545	-0.180	1.574	-7241.451	**1.000**	0.570	0.232	-2.764	12.897

Iberus	-67114.284	0.000	0.494	0.076	-68199.060	0.000	0.556	-0.041	0.416	-68241.331	**1.000**	0.559	0.014	-0.133	1.465
Kovacsia kovacsii	-2640.401	0.000	0.886	0.069	-3045.919	**0.746**	0.978	-0.007	0.500	-3043.762	0.254	0.978	-0.025	0.815	-1.352
Lozekia deubeli	-1249.678	0.000	0.180	0.064	-1244.862	0.000	0.177	0.082	-0.101	-1276.530	**1.000**	0.332	0.595	-7.877	29.926
Marmorana	-4117.958	0.000	0.761	0.231	-4351.121	**0.922**	0.836	-0.103	1.391	-4346.192	0.078	0.837	-0.146	1.839	-1.236
Natalina	-13051.413	0.000	0.718	0.291	-13337.184	0.000	0.756	0.016	0.942	-13424.751	**1.000**	0.767	-0.335	3.719	**-6.389**
Oreohelix	-23392.281	0.000	0.817	0.226	-23867.435	0.000	0.841	-0.020	1.389	-24094.856	**1.000**	0.851	0.210	-2.326	13.717
Partula	-86872.800	0.000	0.220	0.066	-90169.073	0.000	0.422	-0.130	1.043	-92925.391	**1.000**	0.550	0.258	-3.529	14.828
Partulina	-3875.917	0.000	0.955	0.388	-4057.209	0.416	0.967	0.209	0.569	-4057.886	**0.584**	0.968	0.145	1.034	**-0.903**
Paryphanta busbyi	-1219.751	**0.893**	0.551	0.080	-1215.208	0.092	0.547	0.066	0.497	-1211.566	0.015	0.547	-0.006	7.455	-175.513
Placostylus	-1292.365	0.000	0.785	0.409	-1527.328	0.001	0.916	-0.002	1.398	-1540.484	**0.999**	0.922	-0.285	3.702	**-4.557**
Pupilla	-377.650	0.000	0.774	0.217	-447.735	0.228	0.940	-0.101	1.688	-450.169	**0.772**	0.946	-0.324	4.556	**-9.389**
Satsuma	-115192.520	0.000	0.750	0.241	-120412.484	**0.971**	0.815	-0.069	1.136	-120405.492	0.029	0.815	-0.083	1.265	-0.318
Sericata	-2324.352	0.000	0.495	0.132	-2355.735	**0.945**	0.544	-0.052	0.767	-2350.066	0.055	0.543	-0.019	0.459	0.792
Solatopupa	-628.916	0.039	0.633	0.231	-635.123	**0.872**	0.666	0.003	1.005	-630.557	0.089	0.663	0.040	0.606	1.172
Succinea	-13485.292	0.000	0.846	0.258	-14114.505	0.001	0.887	-0.001	1.189	-14128.408	**0.999**	0.889	-0.108	2.233	**-2.696**
Trochulus	-30210.701	0.000	0.356	0.115	-30446.626	0.000	0.388	-0.011	0.542	-30489.761	**1.000**	0.394	-0.152	1.910	**-3.492**
Vertigo	-17197.753	0.000	0.704	0.235	-21976.852	0.447	0.954	-0.155	1.806	-21977.273	**0.553**	0.955	-0.133	1.528	0.864
Wainuia	-2065.305	**0.915**	0.107	0.045	-2060.410	0.079	0.106	0.072	-0.175	-2055.215	0.006	0.104	0.123	-1.015	3.653
Xerocrassa	-61872.366	0.000	0.447	0.079	-65269.816	0.000	0.646	-0.108	0.685	-65884.234	**1.000**	0.674	0.056	-0.753	3.562
COX2															
Agathylla	-1758.124	0.318	0.524	0.390	-1759.412	**0.605**	0.531	0.165	0.438	-1755.288	0.077	0.532	0.406	-0.755	1.705
Codringtonia	-13311.246	0.000	0.547	0.384	-18353.908	0.000	0.901	-0.437	2.440	-20647.498	**1.000**	0.950	0.259	-2.865	9.830

Gnarosophia bellendekerensis	-6176.902	0.000	0.713	0.220	-6952.447	0.000	0.874	-0.082	1.068	-7021.063	**1.000**	0.884	0.097	-0.690	4.265
Sphaerospira	-12204.769	0.000	0.689	0.227	-13504.400	0.000	0.841	-0.104	1.020	-13813.910	**1.000**	0.864	0.207	-1.291	4.633
Cytb															
Euhadra	-10783.874	0.000	0.702	0.417	-11961.056	0.000	0.832	-0.238	1.720	-12251.642	**1.000**	0.854	0.387	-2.210	7.015
Pupilla	-175.962	0.000	0.939	0.646	-278.441	0.004	0.997	-0.026	1.996	-289.685	**0.996**	0.998	-0.277	5.110	**-7.467**
ND1															
Arion	-3411.806	0.000	0.923	0.853	-3680.778	**0.884**	0.945	0.374	0.719	-3676.723	0.116	0.945	0.473	0.348	0.386
Euhadra	-9704.688	0.000	0.880	0.694	-10943.746	0.000	0.934	0.119	1.140	-10968.041	**1.000**	0.935	0.299	0.302	1.092
ND4L															
Euhadra	-9149.516	0.000	0.868	0.636	-9350.487	**0.855**	0.881	0.332	0.612	-9346.943	0.145	0.881	0.222	1.141	-0.720
ATP8															
Albinaria	-2436.027	0.000	0.894	0.785	-2545.044	0.000	0.916	0.256	1.864	-2562.624	**1.000**	0.920	-0.119	5.754	**-10.261**
Cepaea nemoralis	-3368.639	0.000	0.957	0.376	-3377.405	0.000	0.958	0.193	0.770	-3411.177	**1.000**	0.961	0.706	-4.304	13.177

References cited

1. Kotsakiozi P, Parmakelis A, Giokas S, Papanikolaou I, Valakos ED (2012) Mitochondrial phylogeny and biogeographic history of the Greek endemic land-snail genus *Codringtonia* Kobelt 1898 (Gastropoda, Pulmonata, Helicidae). Mol Phylogenet Evol 62: 681-692.
2. Winnepenninckx B, Backeljau T, De Wachter R (1993) Extraction of high molecular weight DNA from molluscs. Trends Genet 9: 407.
3. Parmakelis A, Spanos E, Papagiannakis G, Louis C, Mylonas M (2003) Mitochondrial DNA phylogeny and morphological diversity in the genus *Mastus* (Beck, 1837): a study in a recent (Holocene) island group (Koufonisi, south-east Crete). Biol J Linn Soc 78: 383-399.
4. Folmer O, Black M, Hoeh W, Lutz R, Vrijenhoek R (1994) DNA primers for amplification of mitochondrial cytochrome c oxidase subunit I from diverse metazoan invertebrates. Mol Mar Biol Biotechnol 3: 294-299.
5. Simon C, Frati F, Beckenbach A, Crespi B, Liu H, et al. (1994) Evolution, weighting, and phylogenetic utility of mitochondrial gene-sequences and a compilation of conserved polymerase chain-reaction primers. Ann Entomol Soc Am 87: 651-701.
6. Hugall A, Moritz C, Moussalli A, Stanisic J (2002) Reconciling paleodistribution models and comparative phylogeography in the Wet Tropics rainforest land snail *Gnarosophia bellendenkerensis* (Brazier 1875). Proc Natl Acad Sci U S A 99: 6112-6117.

7. Giokas S (2000) Congruence and conflict in Albinaria (Gastropoda, Clausiliidae). A review of morphological and molecular phylogenetic approaches. Belg J Zool 130: 95-103.
8. Ronquist F, Huelsenbeck JP (2003) MrBayes 3: Bayesian phylogenetic inference under mixed models. Bioinformatics 19: 1572-1574.
9. Posada D, Crandall KA (1998) MODELTEST: testing the model of DNA substitution. Bioinformatics 14: 817-818.
10. Akaike H (1974) A new look at statistical-model identification. IEEE Trans Automat Contr 19: 716-723.
11. Tamura K, Dudley J, Nei M, Kumar S (2007) MEGA4: Molecular evolutionary genetics analysis (MEGA) software version 4.0. Mol Biol Evol 24: 1596-1599.
12. Kimura M (1980) A simple method for estimating evolutionary rates of base substitutions through comparative studies of nucleotide-sequences. J Mol Evol 16: 111-120.
13. Yang ZH, Nielsen R, Goldman N, Pedersen AMK (2000) Codon-substitution models for heterogeneous selection pressure at amino acid sites. Genetics 155: 431-449.
14. Nielsen R, Yang ZH (1998) Likelihood models for detecting positively selected amino acid sites and applications to the HIV-1 envelope gene. Genetics 148: 929-936.
15. Yang ZH (2007) PAML 4: Phylogenetic analysis by maximum likelihood. Molecular Biology and Evolution 24: 1586-1591.
16. Ladoukakis ED, Zouros E (2001) Direct evidence for homologous recombination in mussel (*Mytilus galloprovincialis*) mitochondrial DNA. Mol Biol Evol 18: 1168-1175.
17. Davison A (2000) The inheritance of divergent mitochondria in the land snail, *Cepaea nemoralis*. J Molluscan Stud 66: 143-147.
18. Thomaz D, Guiller A, Clarke B (1996) Extreme divergence of mitochondrial DNA within species of pulmonate land snails. Proc R Soc Biol Sci Ser B 263: 363-368.
19. Galtier N, Nabholz B, Glemin S, Hurst GDD (2009) Mitochondrial DNA as a marker of molecular diversity: a reappraisal. Mol Ecol 18: 4541-4550.
20. Heath L, van der Walt E, Varsani A, Martin DP (2006) Recombination patterns in aphthoviruses mirror those found in other picornaviruses. J Virol 80: 11827-11832.
21. Salminen M, Martin DP (2009) Detecting and characterizing individual recombination events. In: Lemey P, Salemi M, Vandamme A-M, editors. The phylogenetic handbook A practical approach to phylogenetic analysis and hypothesis testing. 2 ed. Cambridge: University Press. pp. 564-590.
22. Nei M, Gojobori T (1986) Simple methods for estimating the numbers of synonymous and nonsynonymous nucleotide substitutions. Mol Biol Evol 3: 418-426.
23. Jukes TH, Cantor CR (1969) Evolution of protein molecules. In: Munro HN, editor. Mammalian Protein Metabolism. New York.: Academic Press. pp. 21-132.
24. Schwarz G (1978) Estimating the dimension of a model. Ann Stat 6: 461-464.
25. Burnham KP, Anderson DR (2002) Model selection and multimodel inference: a practical information-theoretic approach: Springer, New York.
26. Diniz-Filho JAF, Rangel TFLVB, Bini LM (2008) Model selection and information theory in geographical ecology. Glob Ecol Biogeogr 17: 479-488.
27. R Development Core Team (2011) R: A language and environment for statistical computing. Vienna, Austria: R Foundation for Statistical Computing.

***Cyc* sequence data (<200 bp) not deposited in GenBank**

***Albinaria* species *Cyc* sequences (Fasta alignment)**
>AaDia1
NNNNNNNCAGTGCCACACTGTAGAGGCTGGTGGAAAACACAAGACTGGTCCCAATCTCCATGGCCTTATTGGTCGTAAGA
CTGGTCAGGCACCTAATTTTGCTTATACTGAAGCAAACAAGAGCAAAGGTATTACATGGACAAGGGAAACCCTCTTTGAA
TATTTAAAAAATCCCAAGAAATACATCCCCGGCACNNNNNNN
>AaDia2
NNNNNNNNNNNNNNNNNNNNNNNNNNNTGGTGGAAAACACAAGANTGGTCCCAATCTCCATGGCCTTATTGGTCGTAAGA
CCGGTCAGGCACCTAATTTTGCTTATACTGAAGCAAACAAGAGCAAAGGTATTACATGGACAAGGGAAACCCTTTTTGAA
TATTTAAAAAATCCCAAGAAATACATCCCCGGCACCNNNNNN
>AaDia3
NNNNNNNNNNNNNNNNNNNNNNNNNNNTGGTGGAAAACACAAGACTGGTCCCAATCTCCATGGCCTTATTGGTCGTAAGA
CCGGTCAGGCACCTAATTTTGCTTATACTGAAGCAAACAAGAGCAAAGGTATTACATGGACAAGGGAAACCCTTTTTGAA
TATTTAAAAAATCCCAAGAAATACATCCCCGGCACCAANNNN
>AaDia4
NNNNNNNNNNNNNNNNNNNNNNNNNNNTGGTGGAAAACACAAGACTGGTCCCAATCTCCATGGCCTTATTGGTCGTAAGA
CCGGTCAGGCACCTAATTTTGCTTATACTGAAGCAAACAAGAGCAAAGGTATTACATGGACAAGGGAAACCCTTTTTGAA
TATTTAAAAAATCCCAAGAAATACATCCCCGGCACCAANNNN
>AaKast3
NNNNNNNNNNNNNNNNNNNNNNNNNNTTGGTGGAAAACACAAGACTGGTCCCAATCTCCATGGCCTTATTGGTCGTAAGA
CCGGTCAGGCACCTAATTTTGCTTATACTGAAGCAAACAAGAGCAAAGGTATTACATGGACAAGGGAAACCCTTTTTGAA
TATTTNNNNNNNNNNNNNNNNNNNNNNNNNNNNNNNNNNNNN
>AbHel3
NNNNNNNNNNNNNNNNNNNNNNNNNNTTGGTGGAAAACACAAGACTGGTCCCAATCTCCATGGCCTTATTGGTCGTAAGA
CCGGTCAGGCACCTAATTTTGCTTATACTGAAGCAAACAAGAGCAAAGGTATTACATGGACNAGGGAAACCCTTTTTGAA
TATTTAAAAAATCCCAAGAAATACATCCCCGAAANNNNNNNN
>Ac150_1
NNNNNNNNNNNNNNNNNNNNNNNNNNTTGGTGGAAAACACAAGACTGGTCCCAATCTCCATGGCCTTATTGGTCGTAAGA
CCGGTCAGGCACCTAATTTTGCTTATACTGAAGCAAACAAGAGCAAAGGTATTACATGGACAAGGGAAACCCTTTTTGAA
TATTTAAAAAATCCCAAGAAATACATCCCCGANNNNNNNNNN
>Ac358_1
NNNNNNNNNNNNNNNNNNNNNNNNNNTTGGTGGAAAACACAAGACTGGTCCCAATCTCCATGGCCTTATTGGTCGTAAGA
CNGGTCANGCACCTAATTTTGCTTATACTGAAGCAAACAAGAGCAAAGGTATTACATGGACAACGNAAACCCTTTTTGAA

TATTTAAAANATCCCAAGAAATACATCCCCGANNNNNNNNNNN
>Ac359_1
NNNNNNNNNNNNNNNNNNNNNNNNNNTTGGTGGAAAACACAAGACTGGTCCCAATCTCCATGGCCTTATTGGTCGTAAGA
CCGGTCAGGCACCTAATTTTGCTTATACTGAAGCAAACAAGAGCAAAGGTATTACATGGACAAGGGAAACCCTTTTTGAA
TATTTAAAANATCCCAAGAAATACATCCCCGANNNNNNNNNNN
>Ac360_1
NNNNNNNNNNNNNNNNNNNNNNNNNNTTGGTGGAAAACACAAGACTGGTCCCAATCTCCATGGCCTTATTGGTCGTAAGA
CCGGTCAGGCACCTAATTTTGCTTATACTGAAGCAAACAAGAGCAAAGGTATTACATGGACAAGGGANACCCTNTTTGAA
TATTTAAAAAATCCCAAGAAATACATCCCCGANNNNNNNNNNN
>AcKosCas3
NNNNNNNNNNNNNNNNNNNNNNNNNNTTGGTGGAAAACACAAGACTGGTCCCAATCTCCATGGCCTTATTGGTCGTAAGA
CCGGTCAGGCACCTAATTTTGCTTATACTGAAGCAAACAAGAGCAAAGGTATTACATGGACAAGGGAAACCCTTTTTGAA
TATTTAAAAAATCCCAAGAAATACATCCCCGANNNNNNNNNNN
>Al150_1
NNNNNNNNNNNNNNNNNNNNNNNNNNTTGGTGGAAAACACAAGACTGGTCCCNATNTCCATGGCCTTATTGGTNGTAAGN
CNGGTCAGGCACCTAATTTTGCTTATACTGAAGCAAACAAGAGCAAAGGTATTACATGGACNAGGGAAACCCTTTTTGAA
TATTTANAAAATCCCAAGAAATACATCCCCGNNNNNNNNNNNN
>ApKarp3
NNNNNNNNNNNNNNNNNNNNNNNNNNTTGGTGGAAAACACAAGACTGGTCCCNATNTCCATGGCCTTATTGGTNGTAAGA
CCGGTCAGGCACCTAATTTTGCTTATACTGAAGCAAACAAGAGCAAAGGTATTACATGGACNAGGGANACCCTNTTTGAA
TATTTANAAAATCCCAAGAAATACATCCCCGANNNNNNNNNNN
>AvPar3
NNNNNNNNNNNNNNNNNNNNNNNNNNTTGGTGGAAAACACAAGACTGGTCCCAATCTCCATGGCCTTATTGGTCGTAAGA
CCGGTCAGGCACCTAATTTTGCTTATACTGAAGCAAACAAGAGCAAAGGTATTACATGGACAAGGGAAACCCTNTTTGAA
TATTTAAAAAATCCCAAGAAATACATCCCCGAANNNNNNNNNN
>AvPar4
NNNNNNNNNNNNNNNNNNNNNNNNNNNTGGTGGAAAACACAAGACTGGTCCCAATCTCCATGGCCTTATTGGTCGTAAGA
CCGGTCAGGCACCTAATTTTGCTTATACTGAAGCAAACAAGAGCAAAGGTATTACATGGACAAGGGAAACCCTNTTTGAA
TATTTAAAAAATCCCAAGAAATACATCCCCGAANNNNNNNNNN

Codringtonia species _Cyc_ sequences (Fasta alignment)

>Ce_ST6_3
NNNNNNNNNNNNNNNNNNNNNNNNNNNNNNNNNNNNNNNNNNNNNNNNNNNNNNNNNNNNNNGCTTGGTGGCAAACAC
AAGACTGGGCCCAATCTCAGTGGCCTGTTTGGTCGTAAGACTGGTCAAGCACCGGACTTTGCATATACTGAAGCAAAC
AAGAGCAAAGGTATTACATGGACCAGACAGACCCNNNNNNNNNNNNNNNNNNNNNNNNNNNNNNNNNNNNNNNNNNNN
NNNNNNNNNNNNNNNN
>Ceu_Mist5
NNNNNNNNNNNNNNNNNNNNNNAGGTCTTTGTACTAAGATGCCAGCAGTGCCATACTGTGGAACATGGTGGCAAACAT
AAGACTGGTCCCAATCTCAGTGGNNNNNNNNNNNNNNNNNNNNNNNNNNNNNNNNNNNNNNNNNNNNNNNNNNNNNNN
NNNNNNNNNNNNNNNNNNNNNNNNNNNNNNNNNNNNNNNNNNNNNNNNNNNNNNNNNNNNNNNNNNNNNNNNNNNNNN
NNNNNNNNNNNNNNNN
>Cg_MelR5_1
NNNNNNNNNNNNNNNNNNNNNNNNNNNNNNNNNNNNNNNNNNNNNNNNNNNNNNNNNNNGGAGCNNGGTGGCAAACAC
AAGACTGGGCCCAATCTCAATGGCCTGTTTGGTCGTAAGACTGGTCAAGCACCCGGTTTTGCATATACTGAAGCAAAT
AAGAGCAAAGGTATTACATGGACCAGACAGACCCTTTTTGAATATTGGAGAACCCCAAAAAGTACATCCCTGGCACC
AAGATNAANNNNNNNN
>Ch_MEN31_R3
NNNNNNNNNNNNNNNNNNNNNNNNNNNNNNNNNNNNNNNNNNNNNNNNNNNNNNNNNNNGGAGCTTGGTGGCAAACAT
AAGACTGGTCCCAATCTCAGTGGCCTGTTTGGTCGTAAGACTGGTCAAGCACCCGGNTTTGCATNNNNNNNNNNNNNN
NNNNNNNNNNNNTATTACATGGACCAGACAGACCCGTTTTGACTANNTGGAGAACCCAAANANNNNNNNNNNNNNNNN
NNNNNNNNNNNNNNNN
>Ci_Dia4
GGTGATGCTGAGAAGGGCAAGAAGGTCTTTGTACTAAGATGCATGCAGTGCCATTCTGTGGAGCCTGGTGGCAAACAC
AAGACTGGGCCCAATCTCAATGGCCTGTTTGGTCGTAAGACTGGTCAAGCACCGGGCTTTTCATATACTGAAGCAAAT
AAGAGCAAAGGTATTACATGGACCAGGCAGACCCNNNNNNNNNNNNNNNNNNNNNNNNNNNNNNNNNNNNNNNNNNNN
NNNNNNNNNNNNNNNN
>Ch_MEN19_R4_2
NNNNNNNNNNNNNNNNNNNNNNNNNNNNNNNNNNNNNNNNNNNNNNNNNNNNNNNNNNNGGAGCTTGGTGGCAAACAT
AAGACTGGTCCCAATCTCAGTGGCCTGTTTGGTCGTAAGACTGGTCAAGCACCCGGNTTTGCATNNNNNNNNNNNNNN
NNNNNNNNNNNNTATTACATGGACCAGACAGACCCGTTTTGACTANNTGGAGAACCCAAANNNNNNNNNNNNNNNNNN
NNNNNNNNNNNNNNNN
>Cp_The1
NNNNNNNNNNNNNNNNNNNNNNAGGTNTTTGTACTAAGATGCCAGCAGTGCCATACTGTGGAGCCTGGTGGCAAACAC

AAGANNNNNNNNNNNNNNNNNNNNNNNNNNNNNNNNNNNNNNNNNNNNNNNNNNNNNNNNNNNNNNNNNNNNNNNNNN
NNNNNNNNNNNNNNNNNNNNNNNNNNNNNNNNNNNNNNNNNNNNNNNNNNNNNNNNNNNNNNNNNNNNNNNNNNNNNN
NNNNNNNNNNNNNNNN

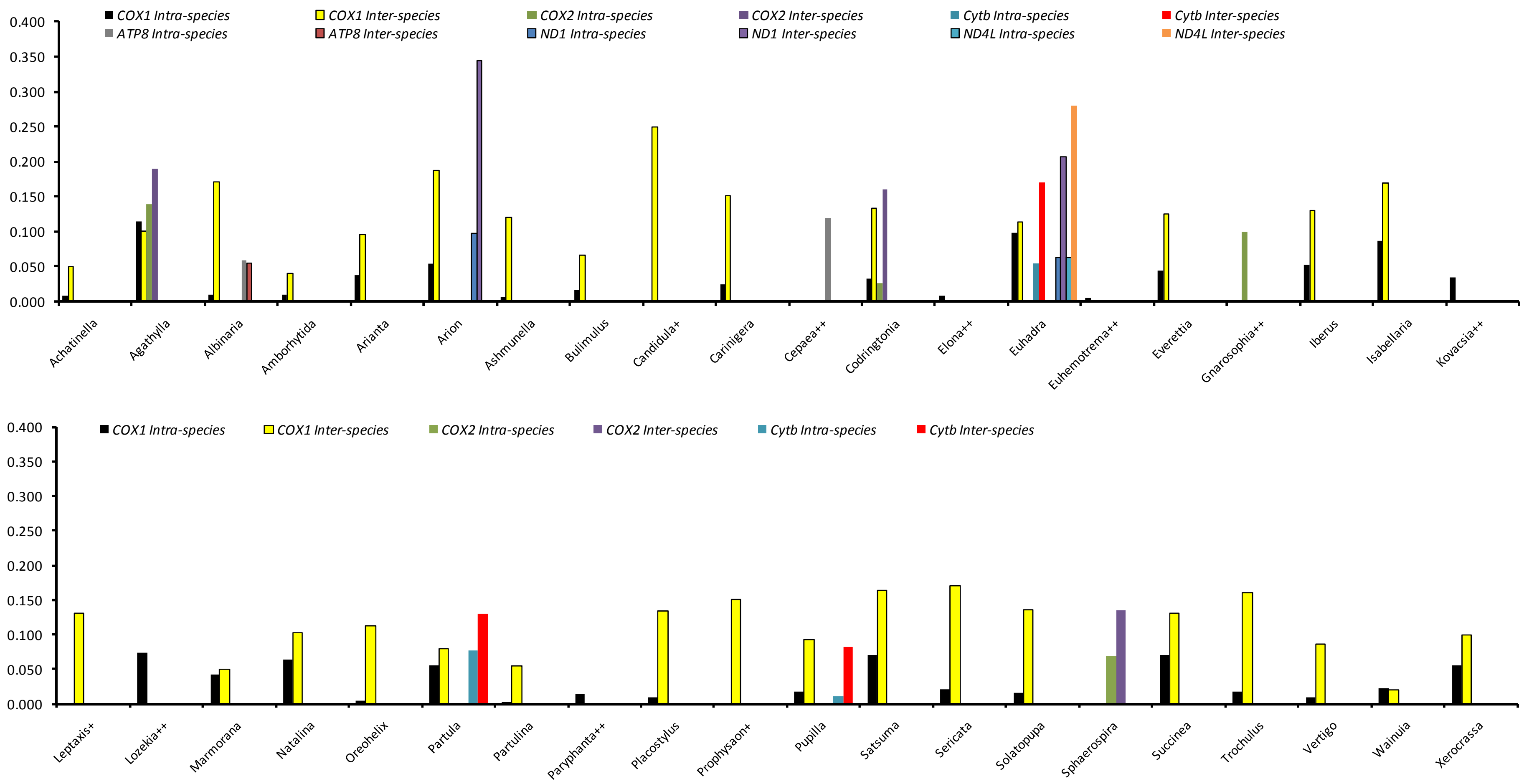

Figure S1. Mean levels of sequence divergence between species (mean inter-species divergence) and within species (mean intra-species divergence) based on the Kimura-2p genetic distance model. The + symbol denotes genera with data limitations that did not permit the calculation of a valid estimate, whereas ++ denotes genera for which only sequences of a single species were available.

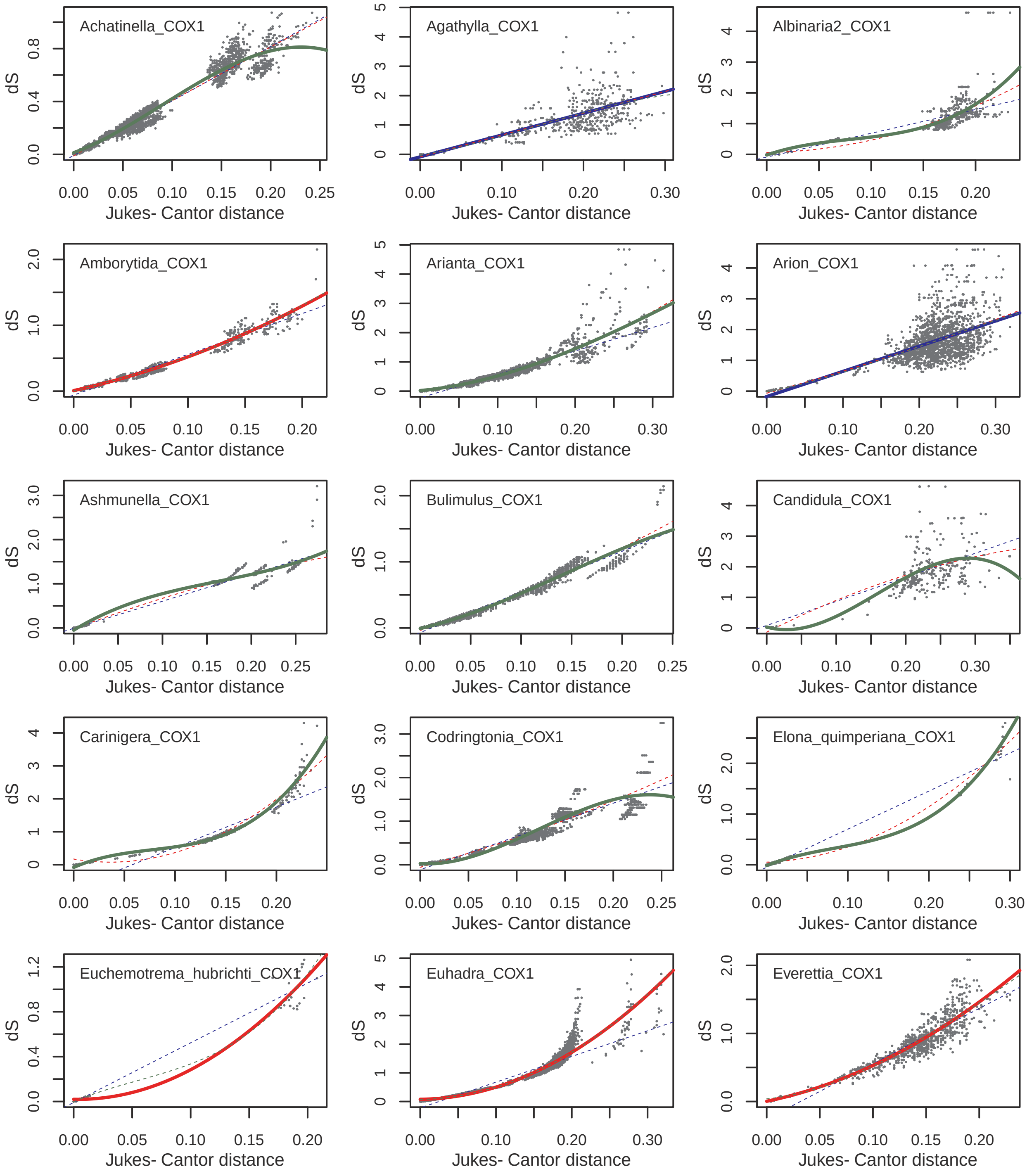

Figure S2a. The dS values as a function of the genetic distance shown using three different models. The best fitted model is in bold line. Blue color corresponds to the linear model, red to the quadratic and green to the cubic model.

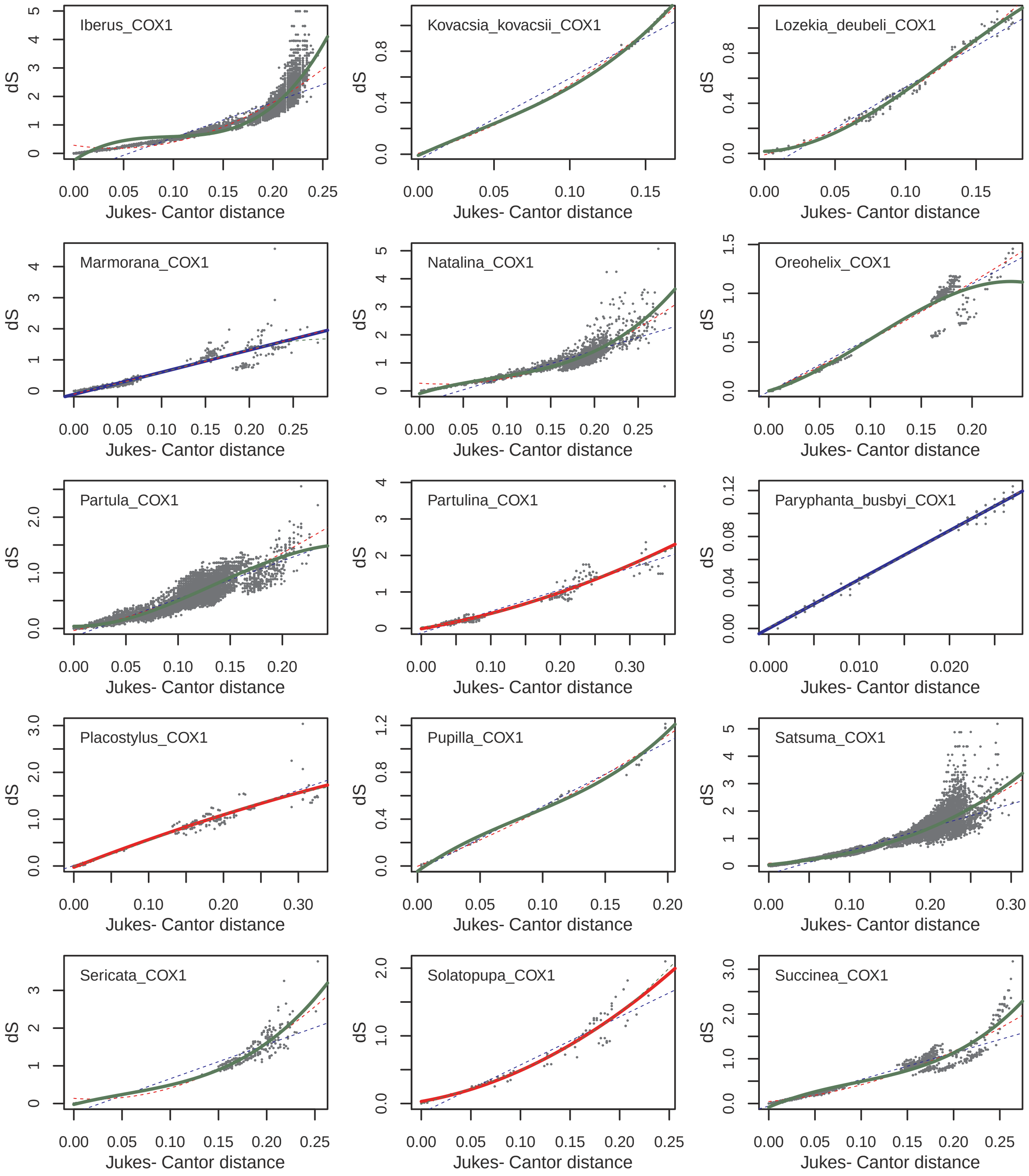

Figure S2b. The dS values as a function of the genetic distance shown using three different models. The best fitted model is in bold line. Blue color corresponds to the linear model, red to the quadratic and green to the cubic model.

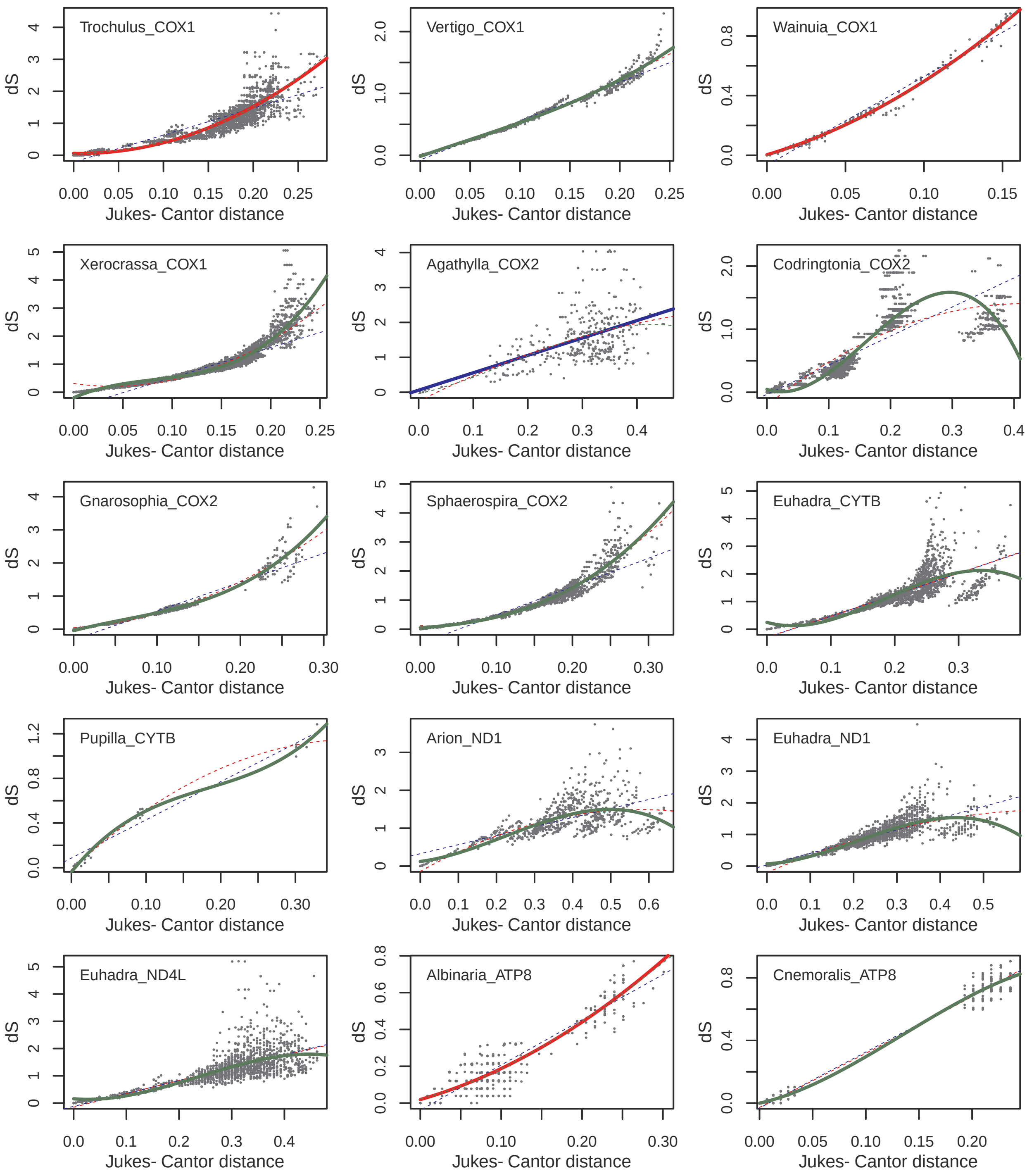

Figure S2c. The dS values as a function of the genetic distance shown using three different models. The best fitted model is in bold line. Blue color corresponds to the linear model, red to the quadratic and green to the cubic model.

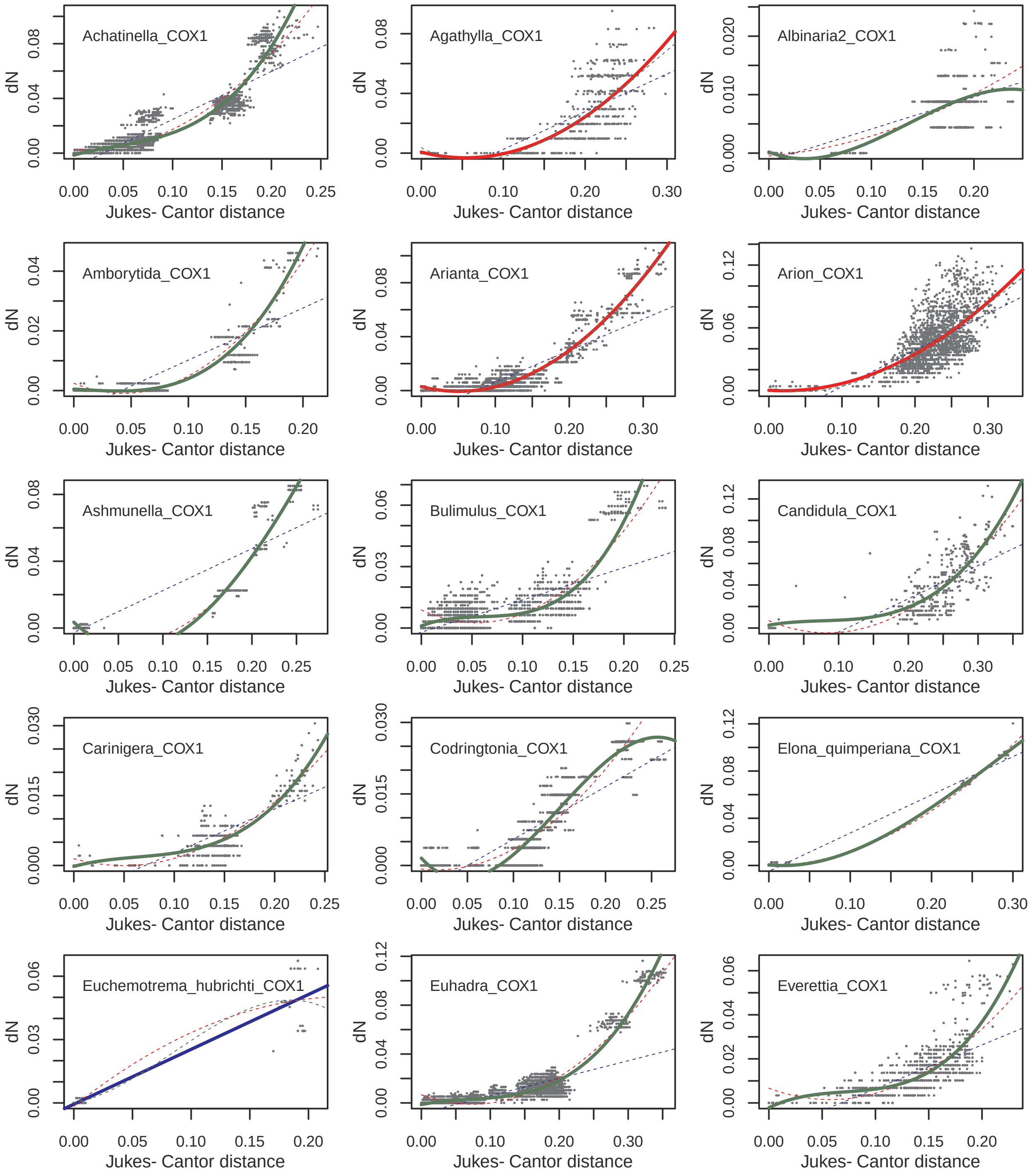

Figure S3a. The dN values as a function of the genetic distance shown using three different models. The best fitted model is in bold line. Blue color corresponds to the linear model, red to the quadratic and green to the cubic model.

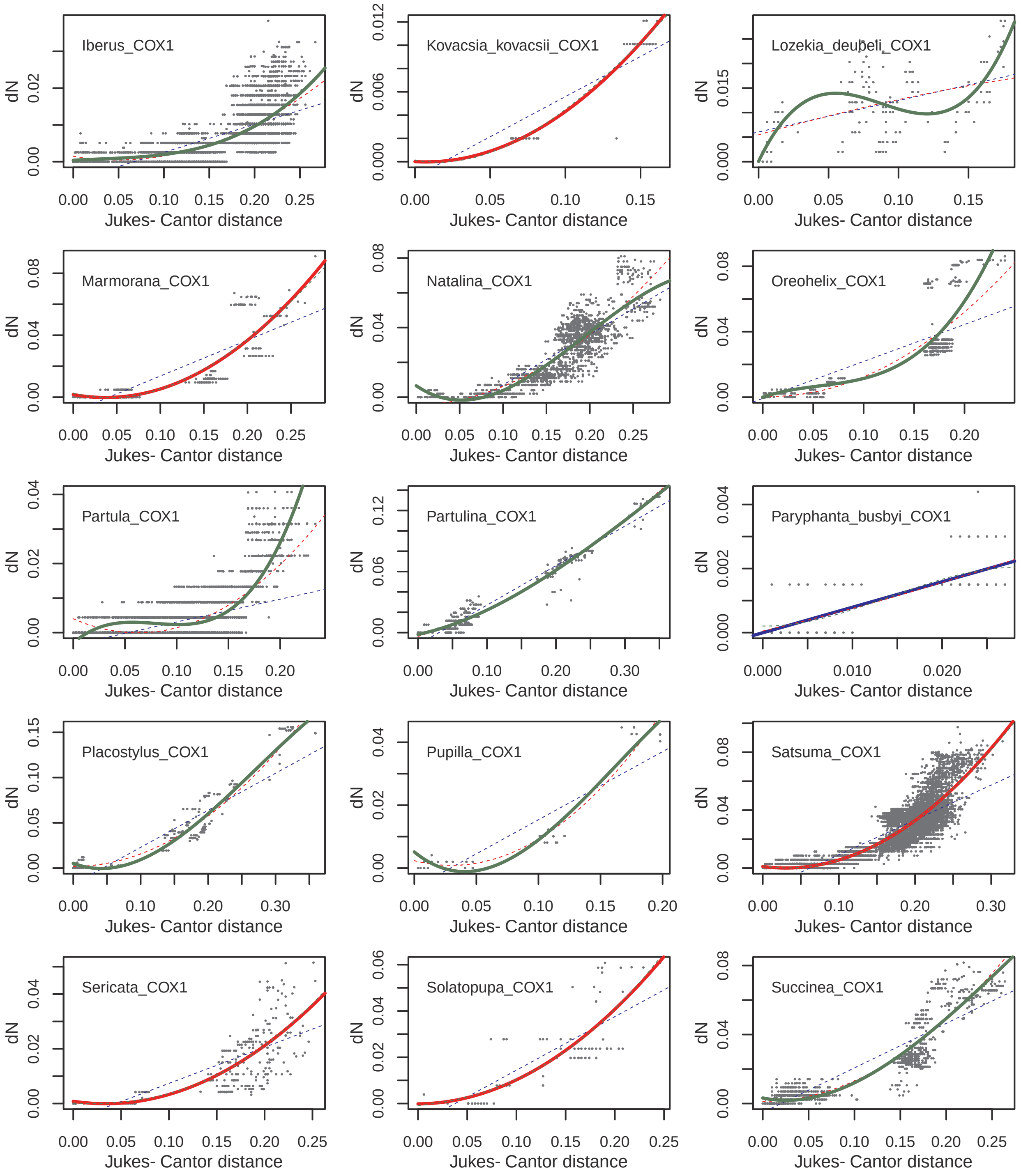

Figure S3b. The dN values as a function of the genetic distance shown using three different models. The best fitted model is in bold line. Blue color corresponds to the linear model, red to the quadratic and green to the cubic model.

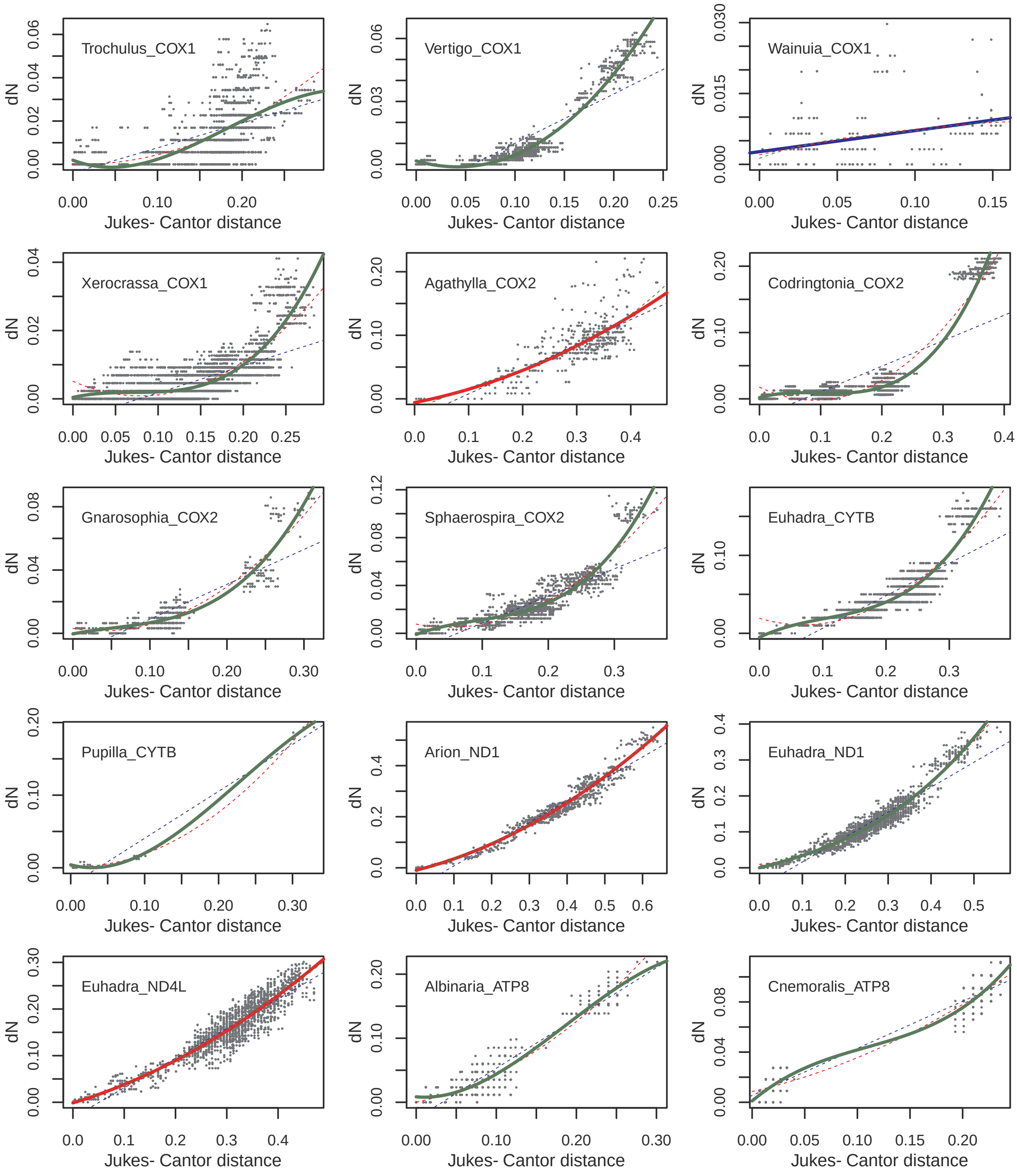

Figure S3c. The dN values as a function of the genetic distance shown using three different models. The best fitted model is in bold line. Blue color corresponds to the linear model, red to the quadratic and green to the cubic model.
